# Development of a multicomponent implementation strategy to reduce upper gastrointestinal bleeding risk in patients using warfarin and antiplatelet therapy, and protocol for a pragmatic multilevel randomized factorial pilot implementation trial

**DOI:** 10.1186/s43058-022-00256-8

**Published:** 2022-01-28

**Authors:** Jacob E. Kurlander, Danielle Helminski, Michael Lanham, Jennifer L. Henstock, Kelley M. Kidwell, Sarah L. Krein, Sameer D. Saini, Caroline R. Richardson, Raymond De Vries, Kenneth Resnicow, Allison Laboon Ruff, David M. Wallace, Elizabeth K. Jones, Linda K. Perry, Jacqueline Parsons, Nghi Ha, Tina Alexandris-Souphis, Dale Dedrick, Elizabeth Aldridge, Geoffrey D. Barnes

**Affiliations:** 1 Department of Internal Medicine, University of Michigan, Ann Arbor, MI USA; 2 Institute for Healthcare Policy and Innovation, University of Michigan, Ann Arbor, MI USA; 3 Veterans Affairs (VA) Ann Arbor Center for Clinical Management Research, Ann Arbor, MI USA; 4 Department of Learning Health Sciences, University of Michigan, Ann Arbor, MI USA; 5 Department of Obstetrics and Gynecology, University of Michigan, Ann Arbor, MI USA; 6 University of Michigan Office of Clinical Informatics, Ann Arbor, MI USA; 7 Health Information Technology and Services, University of Michigan, Ann Arbor, MI USA; 8 Department of Biostatistics, University of Michigan School of Public Health, Ann Arbor, MI USA; 9 Department of Family Medicine, University of Michigan, Ann Arbor, MI USA; 10 Center for Bioethics and Social Sciences in Medicine, University of Michigan, Ann Arbor, MI USA; 11 Department of Health Behavior and Health Education, University of Michigan School of Public Health, Ann Arbor, MI USA; 12 University of Michigan Rogel Cancer Center, Ann Arbor, MI USA; 13 Frankel Cardiovascular Center, Department of Internal Medicine, University of Michigan, Ann Arbor, MI USA

**Keywords:** Implementation science, Patient activation, Nurse facilitation, Warfarin, Anticoagulation, Patient safety, Guideline adherence, Quality of health care, Proton pump inhibitors, Gastrointestinal hemorrhage, Gastroprotection

## Abstract

**Background:**

The concomitant use of anticoagulant and antiplatelet medications increases the risk of upper gastrointestinal (GI) bleeding. Two underused evidence-based practices (EBPs) can reduce the risk: de-prescribe unnecessary antiplatelet therapy or initiate a proton pump inhibitor. We describe the development of a multicomponent intervention to increase use of these EBPs in patients treated with warfarin and followed by an anticoagulation monitoring service (AMS), and the design of a pilot pragmatic implementation trial.

**Methods:**

A participatory planning group iteratively used Implementation Mapping and the Multiphase Optimization Strategy to develop implementation strategies and plan the trial. Informed by qualitative interviews with patients and clinicians, we drew on several implementation science theories, as well as self-determination theory, to design interventions. For patients, we developed an activation guide to help patients discuss the EBPs with their clinicians. For clinicians, we developed two electronic health record (EHR)-based interventions: (1) clinician notification (CN) consists of a templated message that identifies a patient as high risk, summarizes the EBPs, and links to a guidance statement on appropriate use of antiplatelet therapy. (2) Clinician notification with nurse facilitation (CN+NF) consists of a similar notification message but includes additional measures by nursing staff to support appropriate and timely decision-making: the nurse performs a chart review to identify any history of vascular disease, embeds indication-specific guidance on antiplatelet therapy in the message, and offers to assist with medication order entry and patient education. We will conduct a pilot factorial cluster- and individual-level randomized controlled trial with a primary objective of evaluating feasibility. Twelve clinicians will be randomized to receive either CN or CN+NF for all their patients managed by the AMS while 50 patients will be individually randomized to receive either the activation guide or usual care. We will explore implementation outcomes using patient and clinician interviews along with EHR review.

**Discussion:**

This pilot study will prepare us to conduct a larger optimization study to identify the most potent and resource conscious multicomponent implementation strategy to help AMSs increase the use of best practices for upper GI bleeding risk reduction.

**Trial registration:**

ClinicalTrials.gov NCT05085405. Registered on October 19, 2021—retrospectively registered.

**Supplementary Information:**

The online version contains supplementary material available at 10.1186/s43058-022-00256-8.

Contributions to the literature

- This project provides insights into how Implementation Mapping and the Multiphase Optimization Strategy were used iteratively to design and plan the evaluation of multilevel implementation strategies to reduce upper gastrointestinal bleeding risk in anticoagulated patients.
- The implementation strategies take a clinically nuanced approach, prompting clinicians to consider two evidence-based practices, one to de-implement anti-platelet therapy and the other to initiate gastroprotection with proton pump inhibitors.
- The pilot trial described will use a factorial design to evaluate two clinician-facing implementation interventions, as well as patient activation, a strategy that has been widely advocated for use in patient safety initiatives.

## Background

Increasing numbers of patients in the United States (US) are prescribed oral anticoagulants to treat or prevent a range of thromboembolic conditions, including stroke and venous thromboembolism [1]. The main risk with anticoagulant use is major bleeding, occurring 40% of the time in the gastrointestinal tract [2, 3]. Many patients prescribed anticoagulants are co-prescribed an antiplatelet drug (aspirin or a thienopyridine), typically for prevention or treatment of comorbid atherosclerotic cardiovascular disease. These patients on anticoagulant-antiplatelet therapy are at particularly high risk for major bleeding from the gastrointestinal tract and elsewhere [4–6]. In fact, anticoagulants are associated with more emergency department visits for medication harms than any other drug class, and the risk of complications increases with the use of antiplatelet therapy [7]. In an observational study of patients prescribed warfarin without an indication for antiplatelet therapy, antiplatelet drug use increased the risk of major bleeding (5.7% vs. 3.3%), emergency department visits for bleeding (13.3% vs. 9.8%), and hospitalizations for bleeding (8.1% vs. 4.1%), but did not reduce the rate of thrombosis [6].

Two medication-optimization strategies can substantially reduce bleeding risk for patients prescribed anticoagulant-antiplatelet therapy: (1) discontinue antiplatelet therapy in patients for whom it is inappropriate, while continuing the anticoagulant. Based on recent clinical trial data, the indications for anticoagulant-antiplatelet therapy are increasingly narrow, and most patients prescribed anticoagulants should only use antiplatelet drugs for a limited time after acute coronary syndrome, coronary stenting, or other vascular procedures [8, 9]; (2) use a proton pump inhibitor (“PPI gastroprotection”) when anticoagulant-antiplatelet therapy is truly indicated, as recommended by professional guidelines [8, 10]. A meta-analysis showed PPIs reduce the risk of upper gastrointestinal bleeding (UGIB) by up to 79% in patients using aspirin or non-steroidal anti-inflammatory drugs (NSAIDs) [11]. Both evidence-based practices (EBPs) are underused. In an observational study of six anticoagulation clinics, 45% of patients prescribed warfarin were co-prescribed an antiplatelet drug [12]. Of these, 44% had no identifiable indication for antiplatelet therapy, and 36% were appropriately prescribed antiplatelet therapy but without a PPI.

There are multiple barriers to use of these EBPs. We have found that clinicians may lack knowledge of indications for PPI gastroprotection, have inadequate time or prioritization, and lack “ownership,” since many patients are co-managed by a primary care provider (PCP) and a subspecialist (typically a cardiologist) [13, 14]. Many clinicians may be reluctant to discontinue a medication initiated by someone else [15] or may be prepared to assess use of one of the EBPs but not the other, which may lead to suboptimal care. Clinicians may also have concerns about provoking a cardiovascular event when deprescribing anti-platelet drugs and about possible PPI adverse effects when initiating a PPI [14].

There is a critical need for implementation strategies to improve medication optimization for UGIB risk reduction in patients prescribed anticoagulant-antiplatelet therapy. Importantly, to ensure the most appropriate care, any implementation strategy should assist clinicians in considering which of the two strategies is most appropriate for an individual patient and should target the specialist most capable of deciding, while attending to the need for coordinated care.

### Clinician-level interventions

Previously tested one- or two-component clinician-facing interventions aimed at improving use of PPI gastroprotection (including decision support tools, electronic alerts, audit and feedback, and clinician education) have had limited success [16–19]. Several European studies that have tested multicomponent interventions involving professional education, incentive payments, clinician feedback, and pharmacist support have effectively reduced the proportion of high-risk patients without gastroprotection (odds ratios 0.55–0.72) [16, 20, 21]. However, these complex interventions are resource intensive and challenging to implement in the fragmented US healthcare system. Furthermore, in multicomponent intervention studies that have been successful, it is often unclear which intervention component(s) were active and which were inert. Without such information, potential implementers face the risk of expending limited resources on ineffective intervention components.

### Patient activation

Patient activation has been equated to “understanding one’s role in the care process and having the knowledge, skill, and confidence to manage one’s health and health care” [22]. There have been limited efforts to activate patients to enhance the quality of their care in this clinical domain [23]. Engaging patients to promote medication safety has been advocated by the National Quality Forum [24], the Agency for Health Research and Quality, and World Health Organization, which described patients and families as an “untapped resource” in the effort to promote patient safety [25]. Studies in other domains have shown that even brief interventions meant to enhance patient knowledge and activation in a focused clinical context can improve care delivery. For example, in one randomized trial, a mailed activation letter was successful in prompting patients to discuss medication changes to address inadequate blood pressure control [26]. In another randomized trial, a low-literacy patient education tool quadrupled the rate at which patients and clinicians discussed pneumococcal vaccination and increased the rate of vaccination by five times [27]. The use of written or digital patient engagement tools is appealing because once developed, they are inexpensive to use and scalable.

For reducing upper GI bleeding risk, few patient-facing interventions have been rigorously evaluated. A prior uncontrolled study examined use of a pharmacist-delivered educational intervention (education and a written report to share with the clinician) for patients at high-risk for GI complications from NSAIDs [28]; for patients obtaining prescription NSAIDs, the pharmacist also reached out to the prescriber if needed. However, this study did not report the results for the subgroup of patients who only received the written report. A randomized trial (SPACE) evaluated a multicomponent intervention that includes clinical practice audits to identify high-risk patients using NSAIDs or antiplatelet drugs, outreach visits from clinical pharmacists, and an informational mailing to selected patients recommending that they discuss medication changes with their clinicians [23, 29]. The trial (yet unpublished) will evaluate the effect of the multicomponent intervention overall. A third study described pharmacists’ use of an online patient portal to identify patients at increased risk for GI bleeding, to whom letters were sent recommending discussion with their clinicians. However, the study did not report the effectiveness of the intervention [30].

Despite the critical importance of involving patients in patient safety efforts, undoubtedly not all patients will be able to successfully engage the health care system to improve the quality of care, nor should they be held responsible for this. In fact, some have criticized efforts to engage patients in error prevention as “shifting responsibility” to patients [31]. Patient engagement materials often overlook patients’ perspectives, fail to address what clinicians can do to ensure patient safety, and may place patients in an uncomfortable position of challenging the role of the clinician [31]. It therefore stands to reason that patients should be involved in the process of developing such tools and should not be held solely responsible for improving quality of care.

### The multiphase optimization strategy (MOST)

Because of uncertainty about which clinician-level interventions are effective, how they would function in a US health system (where incentive payments, for example, may not be possible), their interaction effects, and the benefits of patient engagement, we drew upon the MOST framework to develop and identify effective intervention strategies. MOST is a novel approach to designing, optimizing, and evaluating multicomponent interventions for maximal effectiveness using principles of engineering and manufacturing [32]. In the past, most multicomponent interventions were assembled on a theoretical basis and then tested in a single randomized controlled trial (RCT). MOST includes three stages. In the “screening” (or “preparation”) phase, candidate intervention components with high potential are identified, often by conducting brief trials, or identifying promising strategies from previous trials. In the second “refining” or “optimization” phase, interventions that passed the screening phase are included in randomized experiments to evaluate their individual effects; other features such as optimal dosage, interaction effects, or heterogeneity of treatment effects, can also be explored. The “refining phase” often relies on highly efficient designs, such as factorial (or fractional factorial) trials. Finally, in a “confirming phase,” results of the first two phases are used to assemble an optimized intervention, which is finally tested, typically in an RCT. MOST has the advantage of developing multicomponent interventions in which all components are (ideally) effective. This feature is desirable from a resource use perspective, which is critical for interventions that may be widely disseminated.

### Study purpose

Our dual purposes in this manuscript are to describe (1) our use of implementation mapping to systematically develop patient- and clinician-facing interventions to increase use of the medication optimization EBPs, and (2) the design for a pilot pragmatic implementation trial to explore foremost the feasibility of the study design. This pilot study constitutes the preparation phase of MOST. In a follow-up study, we anticipate conducting a factorial trial that is adequately powered to evaluate the independent and interaction effects of patient and clinician interventions; the pilot trial described herein constitutes a miniature version of the anticipated design of this second “refining phase” of MOST.

## Methods

We adhered to the Standards for Reporting Implementation Studies (StaRI) Statement (supplement 1) in this manuscript [33].

### Development of implementation strategies with implementation mapping

Implementation mapping (IM) is an iterative 5-step approach that guides the process of planning, selecting, and evaluating implementation strategies for health promotion interventions (Table 1) [34]. Implementation mapping is derived from intervention mapping, a broader framework for the systematic and theory-based development and implementation of multi-level health interventions. An implementation framework, instead of an intervention development framework, was used for this project since the EBPs (appropriate use of antiplatelet therapy and PPI gastroprotection) are clear—the gap is translating this knowledge into practice. This section describes how we executed each step of the IM process, which encompasses adoption, implementation, and maintenance.

**Table 1:** Implementation mapping steps

Step 1. Conduct a needs and assets assessment and identify adopters and implementers	
Step 2. Identify adoption and implementation outcomes, performance objectives, and determinants; create matrices of change	
Step 3. Choose theoretical methods; select or create implementation strategies	
Step 4. Produce implementation protocols and materials	
Step 5. Evaluate implementation outcomes	

#### Task 1: Conduct an implementation needs assessment and identify adopters and implementers

##### Identification of program adopters and formation of the participatory planning group

The IM process took place at Michigan Medicine, a tertiary academic medical center in the Midwest of the United States, which will also be the site of the pilot trial. The implementation strategies were designed for delivery by the anticoagulation management service (AMS) to patients treated with warfarin, an anticoagulant. The clinical leaders of the AMS affirmed the value of addressing UGIB risk at the outset and a willingness to adopt and maintain the intervention developed. The AMS in this study is overseen by a board-certified cardiovascular physician, staffed by nurses, and provides remote services typically by phone; there are no face-to-face encounters.

There are over 3000 AMSs in the United States, typically staffed by nurses and/or pharmacists, and their overarching purpose is to ensure the safe and effective use of warfarin and other oral anticoagulants [35]. Warfarin has a narrow therapeutic index and requires frequent monitoring of blood tests to ensure proper dosing; under-dosing increases the risk of thromboembolic phenomenon, whereas over-dosing increases the risk of hemorrhage. Some AMSs provide services in face-to-face visits, and others through remote visits, typically phone calls [36]. AMSs often provide adjunctive services to ensure the safe use of warfarin, including education on foods and drugs likely to interfere with warfarin metabolism, and education on how to manage minor bleeding events (e.g., nosebleeds). However, AMSs typically do not undertake activities to minimize UGIB risk. In fact, anticoagulation clinics are not routinely staffed by physicians and as such cannot routinely discontinue or initiate medications. It was therefore determined at the outset that clinicians within the broader health system would be responsible for executing the EBPs (medication optimization).

To assist with all steps of the implementation mapping process, we convened a participatory planning group of stakeholders. The group included physicians from gastroenterology (1), cardiovascular medicine (1), and primary care (2), patients with anticoagulation clinic experience (2), a clinical pharmacist from the anticoagulation clinic (1), anticoagulation service nurses (2), a study coordinator (1), and a health IT specialist (1). The group met 19 times between July 2020 and October 2021. The group was involved in each step of the implementation process, including interpreting the results of the needs assessment, choosing implementation outcomes, selecting implementation strategies, developing implementation materials, and planning of the pilot trial.

##### Identification of barriers and facilitators to the use of PPI gastroprotection

We conducted semi-structured interviews with clinicians and patients to understand barriers and facilitators to medication optimization. Methods and results of the clinician interviews, which included cardiologists (*n*=4), primary care providers (*n*=5), vascular surgeons (*n*=3), and gastroenterologists (*n*=3), are described in a separate publication [14]. This qualitative study had a particular focus on dynamics within and across medical specialties that affect use of PPI gastroprotection because patients on anticoagulant-antiplatelet therapy are often co-managed by multiple specialists (typically a cardiologist and PCP). Briefly, we found that while some cardiologists routinely prescribed gastroprotection, most PCPs, gastroenterologists, and vascular surgeons did not. Barriers to gastroprotection mainly related to (1) knowledge, (2) decision processes, and (3) professional role. Cardiologists tended to have greater knowledge of relevant guidelines on when and how to use PPI gastroprotection and were accustomed to managing antithrombotic medications to minimize bleeding risk; however, they often deferred prescribing PPI gastroprotection to other clinicians. For PCPs, prescribing gastroprotection was a low priority relative to other clinical demands; in fact, many PCPs were more focused on stopping PPIs for patients who were using them unnecessarily. In addition, PCPs were often reluctant to discontinue antithrombotic drugs in patients cared for by a cardiologist. We also found unclear delineation of responsibility for prescribing gastroprotection across specialties.

To complement the clinicians’ perspective, we conducted 5 semi-structured interviews with patients who used antithrombotic medications and were not part of the participatory planning group. Details of the methods can be found in Supplement 2. Guided by the Theoretical Domains Framework (TDF) [37], we identified key areas of focus for patients related to knowledge of GI bleeding and risk factors, knowledge of PPIs and their use for bleeding prevention, clinician responsibility for use of bleeding prevention strategies, and barriers to PPI gastroprotection use. Across the board, patients had limited knowledge of upper GI bleeding risk factors, their personal risk, and of the role of PPIs for bleeding prevention. Patients had greater awareness of and concern about bruising or internal bleeding from other anatomic sites:

I have heard [of] internal bleeding potentially; I never thought of my stomach though. I always thought, like, organs.

Multiple patients believed that diet choices (e.g., spicy foods, excess coffee) would increase their risk of GI bleeding. Patients generally felt the doctor who prescribed their antithrombotic drugs should discuss bleeding risks and mitigation strategies with them. However, most found it acceptable if someone else from their care team, such as a pharmacist or nurse, reached out as long as care was being coordinated with their clinicians. While patients generally felt comfortable initiating a conversation about whether they might benefit from a PPI with their clinicians, some felt they did not have the requisite knowledge to do so.

I don’t think I had the knowledge before this interview to ask for [a proton pump inhibitor].

Barriers to PPI gastroprotection primarily related to medication costs, a desire to minimize the number of medications, and long waiting periods to see a specialist.

I want to be taking as few medications as I can, you know, just what I basically need to treat my condition. I don’t want to overdo it with things because I just – that’s just how I am.

##### Assessing prevalence of anticoagulant-antiplatelet therapy and PPI gastroprotection

Using an electronic data extraction from the electronic health record (EHR) in August 2020, we identified 3242 patients prescribed warfarin who had an encounter with the anticoagulation service in the prior 4 months. Among these, 949 (41.4%) also used aspirin and/or a thienopyridine, according to their medication lists. Of these 949 patients on anticoagulant-antiplatelet therapy, 658 (69.3%) were not using a PPI, which equated to 20.3% of all patients followed by the AMS. For patients using aspirin, the rate of PPI use was 29.2%, and for thienopyridine users, 46.8%. In a previous study of a similar population of patients using anticoagulant-antiplatelet therapy, we found the most appropriate medication optimization strategy was discontinuation of antiplatelet therapy for 56% of patients, while PPI was appropriate in the remaining 44% [12].

As part of task 1, we also conducted a literature review (see Supplement 3), which is summarized in the introduction, and evaluated the accuracy of the EHR medication list compared to self-reported use of antiplatelet therapy and PPIs (see Supplement 4), which demonstrated the ability to accurately identify candidates for medication optimization using electronic data.

#### Task 2: Formulation of change objectives

The participatory planning group next enumerated the steps necessary to accomplish medication optimization. These were formulated into “implementation outcomes” specific to each adopter or implementer, tantamount to their implementation goals. Next, for each implementation outcome, we identified “performance objectives,” which are the sub-behaviors necessary to accomplish the implementation outcome. As an example, one implementation outcome was “Patients who are randomized to receive the patient activation intervention will review the activation guide and contact their clinician to discuss their GI bleeding risk and options to reduce this risk,” with performance objectives of “(1) the patient receives, reviews, and comprehends materials sent by mail or portal and (2) the patient contacts their clinician to discuss appropriate strategies for UGIB risk reduction.” See Supplement 5 for the full elaboration of implementation outcomes and performance objectives. In creating these tables, a critical insight was that some sub-behaviors could be performed by either clinicians (and their care teams) or by nurses in the AMS. Such activities included identifying clinical information necessary for decision-making and providing education to patients on changes in medications. This led us to formulate two sets of implementation outcomes and performance objectives, one that assigned a more active role to AMS nurses, and one that assigned a less active role. These differing sets of implementation outcomes led to the elaboration of two distinct clinician-facing intervention strategies—clinician notification (CN), and clinician notification + nurse facilitation (CN+NF), further described below.

While the group felt that ensuring evidence-based care was primarily the responsibility of clinicians, our literature review demonstrated many clinician-facing interventions have had limited success. Therefore, we developed a set of implementation outcomes related to patient engagement in medication optimization, based on the hypothesis that this approach would augment the effectiveness of clinician-facing interventions.

Next, we identified the determinants (barriers) most relevant to each implementation outcome, informed by the prior qualitative interviews with patients and clinicians. For each implementation outcome, we then crossed the performance objectives with the salient barriers to create a “matrix of change objectives,” which show “what has to change in this determinant to bring about the performance objective” [34]. The full set of matrices can be found in Supplement 5. Based on our prior qualitative work, we focused on the determinants of knowledge, professional role, and memory/attention/decision processes for clinicians. For patients, we focused on the determinants of knowledge, skills (e.g., self-efficacy to engage clinicians), and environmental context and resources (e.g., ability to afford medications).

#### Tasks 3 and 4: Selection of theoretical methods, development of intervention strategies, and production of implementation protocols and materials

In this step, we selected the implementation strategies to influence the determinants/barriers. We relied on multiple sources to identify relevant candidate strategies. These included the Expert Recommendations for Implementing Change (ERIC) compilation of implementation strategies [38]; the Behavior Change Wheel [39], which was made easier by the fact that it explicitly links intervention strategies to barriers included in the TDF (see Step 1); a taxonomy of intervention strategies that has been developed for use with Intervention Mapping [40]; and the findings of our literature review. Candidate strategies were discussed in meetings of the participatory planning group to identify those with the greatest potential and best fit to the setting.

All implementation strategies were designed to be delivered by AMS staff, in keeping with the pragmatic design of the study. Of note, the strategies aim to facilitate appropriate use of the EBPs by clinicians; the AMS and its staff could not directly prescribe or discontinue patients’ medications. The theoretical methods selected for each of the implementation interventions are discussed below. For each of the theories used in selecting implementation interventions and translating them into practical interventions, we took into account the pre-conditions under which theory was likely to be effective (“parameters” in the parlance of Implementation Mapping) [40].

**Fig. 1:**
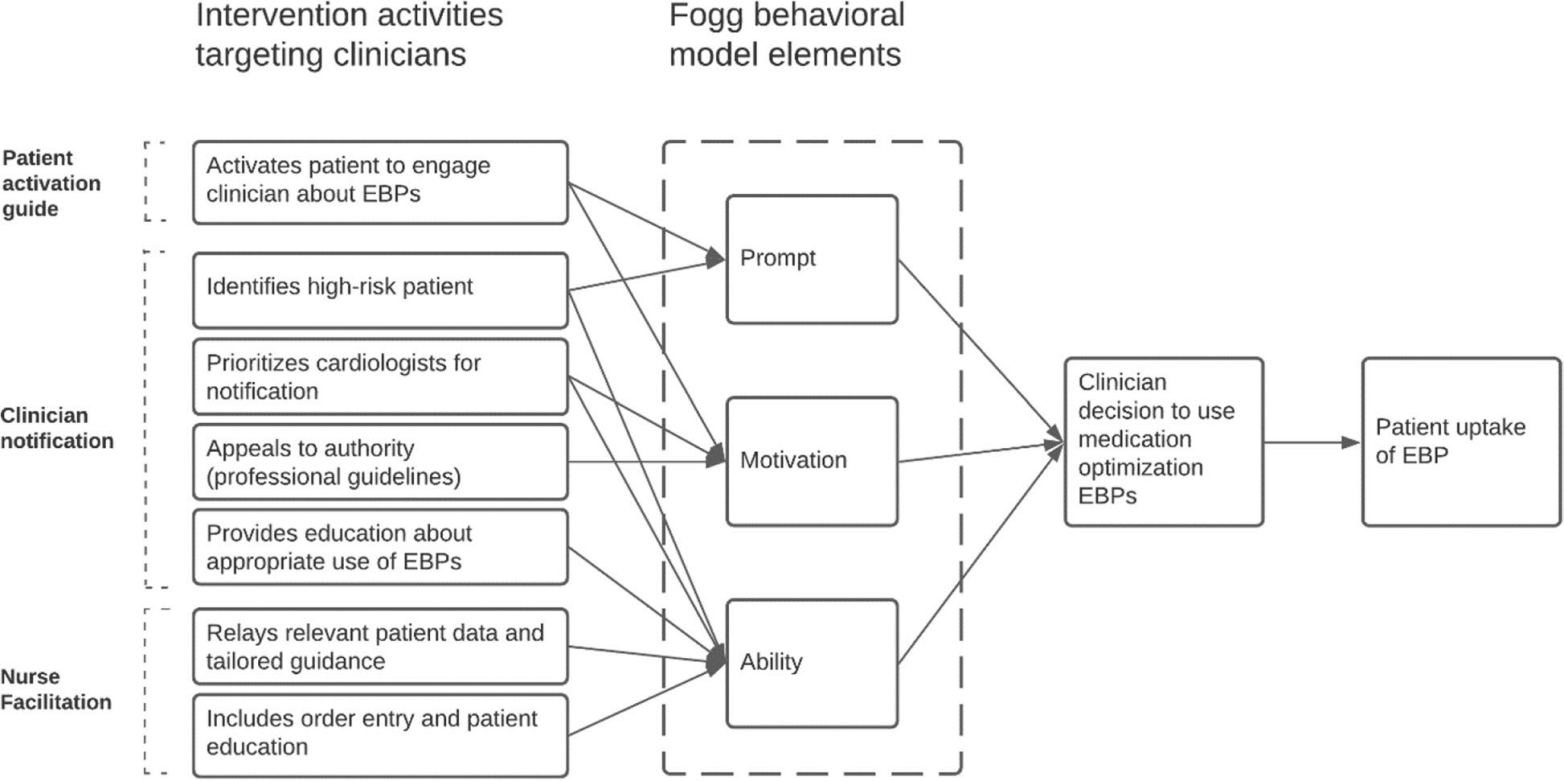
Logic model linking intervention activities to clinician behavior change and patient uptake of medication optimization

Altogether, the implementation strategies developed are responsive to the Fogg Behavioral model, which identifies three pre-conditions necessary to trigger a target behavior: motivation, ability, and a prompt [41]. The logic model by which the activities within each of the clinician- and patient-facing intervention strategies compel clinician prescribing behavior are shown in Fig. 1.

##### Clinician-facing implementation interventions

“*Clinician notification*” (CN): Because clinician knowledge was a prominent barrier to medication optimization, the group elected to notify clinicians in the form of a message sent through the EHR for relevant patients (ERIC strategy: “remind clinicians”). The message identifies the patient as high risk for upper GI bleeding, provides an estimate of upper GI bleeding risk based on population averages (2.8% per year), describes the medication optimization strategies, and contains an intranet link to guideline summaries on appropriate use and duration of antiplatelet therapy in patients using warfarin, compiled as part of the AEGIS project (see Supplement 6; ERIC strategy: “develop and distribute educational materials”). The text of the notification message is shown in Supplement 7. The message also contains a list of EHR “smart texts” that were developed, consisting of templated language that clinicians could quickly include in EHR communications with office staff and other clinicians about medication optimization. An asynchronous notification was chosen instead of an interruptive alert because alerts are more disruptive to workflow, and because they would only be triggered when a clinician was already in a patient’s medical record, which might delay the time to medication optimization.

“*Clinician notification with nurse facilitation*” (CN+NF): This implementation strategy is similar to CN but incorporates additional features to streamline appropriate decision-making based on our finding that time was a major barrier to medication optimization. This strategy leverages the clinical expertise of the AMS nursing staff in three ways: First, they review the medical record of the patient to identify possible indications for antiplatelet therapy (e.g., “CAD with percutaneous coronary intervention [PCI]”), information which is then included in the notification message (ERIC strategy: “Facilitate relay of clinical data to providers”). Second, they provide a concise and tailored summary of guidance on appropriate use of antiplatelet therapy (e.g., “if PCI within the past 12 months, continue antiplatelet therapy, otherwise stop antiplatelet therapy”), avoiding the need for clinicians to review the full 2-page guidance summary on the intranet. Third, once the clinician has decided on the appropriate medication optimization strategy, the message indicates that the nurse can enter order for the PPI and pend it for the clinician’s signature (when relevant), and relay the recommendation to the patient, along with appropriate patient education. Together, these steps would allow the clinician to make an instantaneous decision on medication optimization while minimizing cognitive workload. The content of both the CN and CN+NF messages were reviewed on multiple occasions with the members of the participatory planning group.

In the future, we intend to use two additional ERIC strategies after any modifications to the interventions based on the pilot study—conduct educational meetings with providers and inform local opinion leaders.

##### Patient activation guide

We developed a written activation guide intended to prepare and motivate patients to have an informed discussion with their clinicians about medication optimization and to initiate that discussion if necessary (ERIC strategy of “prepare patients/consumers to be active participants”). The guide was developed for delivery through the electronic patient portal or by mail. We considered the option of having nurses call patients to deliver the intervention verbally, but this would have been prohibitively time-consuming for the clinic staff.

In selecting theories for developing the patient activation guide, we mainly considered using the Health Beliefs Model (HBM) and Self-Determination Theory (SDT), given their prominence in health promotion programs. We identified the role of fear appeals as an important difference between these theories. The HBM model would have led us to emphasize the risk and seriousness of upper GI bleeding. However, evidence suggests that fear appeals may only be effective when patients have high efficacy to enact change [42]. Because we believe many patients lack the efficacy to engage clinicians around medication optimization, we opted to use self-determination theory (SDT) instead [43, 44]. In recognition of the important role of autonomy in SDT, we avoided use of strongly directive language (for example, “You should speak with your doctor”) in favor of more suggestive language (“You may consider speaking with your doctor…”) and presented the two EBPs for medication optimization as “options.” The guide also acknowledged patients’ need for control of the outcome by providing specific language they could use to discuss medication optimization with their clinicians and by providing reassurance that clinicians welcomed their input. Other theoretical methods used in the patient brochure included modelling (vignettes of patients who successfully engaged their clinicians) and persuasive communication (e.g., appeals to medical authority) [45].

The patient activation guide was designed in collaboration with a graphic designer, using Microsoft PowerPoint. The layout and flow were initially modelled off of an activation guide used in a prior successful intervention that activated patients to reduce use of benzodiazepines [46–48]. However, all content for our guide was newly developed. We incorporated principles of patient communication and plain-language as recommended by the Centers for Medicare and Medicaid Services (CMS) [49] and the Centers for Disease Control and Prevention (CDC) [50]. We also included features to enhance ease of information processing [51]. For example, we used informative section headings and pop-out effects, selectively bolding the most important information throughout the guide. The guide was repeatedly revised to ensure text coherence, with logical and clear connections from one section (or sentence) to the next. Few graphic elements were included because they were found to add little information content (e.g., images of pill bottles).

Between March and July 2021, the guide was pre-tested with 12 patients, all of whom self-reported use of an oral anticoagulant. Interviews were completed over Zoom (Zoom Video Communications, Inc. Version: 5.7.0), using a using a think-aloud, user-centered design approach [52]. Details of the approach and the interview guide can be found in Supplement 8. After every 2–3 interviews, the guide was revised based on feedback. We also sought feedback in multiple research forums at the University of Michigan, including the Center for Bioethics and Social Sciences in Medicine (CBSSM) working group and the Michigan Program on Value Enhancement (MPrOVE) Innovation to Implementation (i2i) Lab. The guide was also reviewed multiple times by the participatory planning group.

The final 8-page guide was titled “Did you know a simple change in the medications you take could reduce your risk of bleeding?” (see Supplement 9). One page each provided information about anti-thrombotic drugs, proton pump inhibitors, and the two strategies for medication optimization. We also included two patient vignettes, one for each of the medication optimization strategies, accompanied by images of patients; an FAQ section on gastrointestinal bleeding; and specific instructions and language on how to discuss medication optimization with a clinician. The guide was accompanied by a cover letter addressed to the patient that explained its purpose and was signed by the AMS’s Medical Director.

##### Implementation strategies, protocols, and information systems to support AMS staff

For the patient and clinician-level interventions, nursing protocols were developed (see Supplement 10). Both protocols specified that the notification should always be sent to a cardiologist for patients seen by one within the prior year (with rare exceptions), and otherwise be sent to the clinician of record for the anticoagulation clinic, to whom routine communications are sent. This decision reflects an insight gained from our qualitative interviews that many primary care providers felt lack of ownership of medication optimization for patients who also saw a cardiologist, whom they often preferred make decisions related to antiplatelet therapy and gastroprotection.

To enable anticoagulation staff to carry out the implementation strategies, we developed a quality monitoring system (ERIC strategy), in the form of an EPIC workbench report. Using Boolean logic and EHR data elements, the electronic report identifies patients each day who may benefit from medication optimization. In addition, it summarizes relevant medical data and allows rapid identification of clinicians to whom the clinician-level interventions should be targeted and tracking of delivery of the interventions. By creating a new role for nurses in ensuring medication optimization, these interventions employ the ERIC strategy of “revise professional roles.”

For both the CN and CN+NF messages and the patient activation guide, “smart-text” (templated messages that automatically pull in available encoded data elements) were created that would allow the anticoagulation staff to quickly send the materials through the EHR.

### Pilot trial design (implementation mapping task 5)

We will carry out a pragmatic pilot implementation trial with a cluster- and individual-level randomized factorial design that will primarily assess the feasibility of completing a larger trial with a similar design in the future, and the feasibility of delivering the implementation strategies as intended.

#### Study setting and eligibility

The study will take place at Michigan Medicine, as described above. Nurses from the AMS will deliver the implementation strategies to eligible patients and the clinicians who care for them. Because our study design is multi-level, the study’s inclusion criteria rely on the concept of clinician-patient dyads; a dyad links each patient to a single clinician (hereafter referred to as a “target clinician”). This will result in a one-to-many-relationship between target clinicians and patients. For patients who have seen a cardiologist (with the exception of electrophysiologists) in the prior year, the target clinician is defined as that clinician. Otherwise, the target clinician is defined as the clinician of record with the anticoagulation service. Every patient treated by the anticoagulation service has a designated clinician of record, who may be in any medical specialty, is the prescriber of the warfarin, and receives all routine communications from the AMS. This manner of identifying target clinicians, which gives precedence to cardiologists, is based on our qualitative analysis, as described above. Unlike other cardiologists, cardiologists in the subspecialty of electrophysiology were not given precedence as target clinicians because members of the participatory planning group felt that electrophysiologists were less accustomed to routinely managing anti-platelet therapy and gastroprotection.

Detailed eligibility criteria for clinicians and patients are shown in Table 2. In brief, clinicians will be eligible if they are a target clinician for at least one patient treated by the anticoagulation service. Patients are eligible for participation if they are followed by the AMS, are included in a clinician-patient dyad that contains a participating target clinician, are candidates for medication optimization (i.e., use warfarin and an antiplatelet drug without a PPI), and do not have an allergy or intolerance to a PPI. Eligible patients will be identified using the reporting workbench, described above.

**Table 2:** Eligibility criteria

**Patient eligibility criteria**	
**Inclusion:** • Enrollment with the Michigan Medicine anticoagulation monitoring service • Currently prescribed warfarin with anticipated use for ≥90 days on day 1 of trial enrollment, according to the EHR medication list • Currently prescribed an antiplatelet drug (aspirin, clopidogrel, ticagrelor, or prasugrel) according to the EHR medication list	**Exclusion:** • Age less than 18 • Prescribed a Proton pump inhibitor • Documented intolerance or allergy to Proton pump inhibitor use • Left ventricular assist device • Heart transplant
**Clinician eligibility criteria**	
**Inclusion:** • Practicing cardiologists at Michigan Medicine who in the prior year had a face-to-face or virtual visit with a patient who meets eligibility criteria for this study • Practicing clinicians in any specialty who are designated as the clinician of record with the anticoagulation clinic for a patient who meets eligibility criteria	**Exclusion:** • Cardiologists specializing in electrophysiology who are not the designated clinician of record for at least one patient meeting eligibility criteria

*EHR* electronic health record

#### Study design, sample size, and aims

**Fig. 2:**
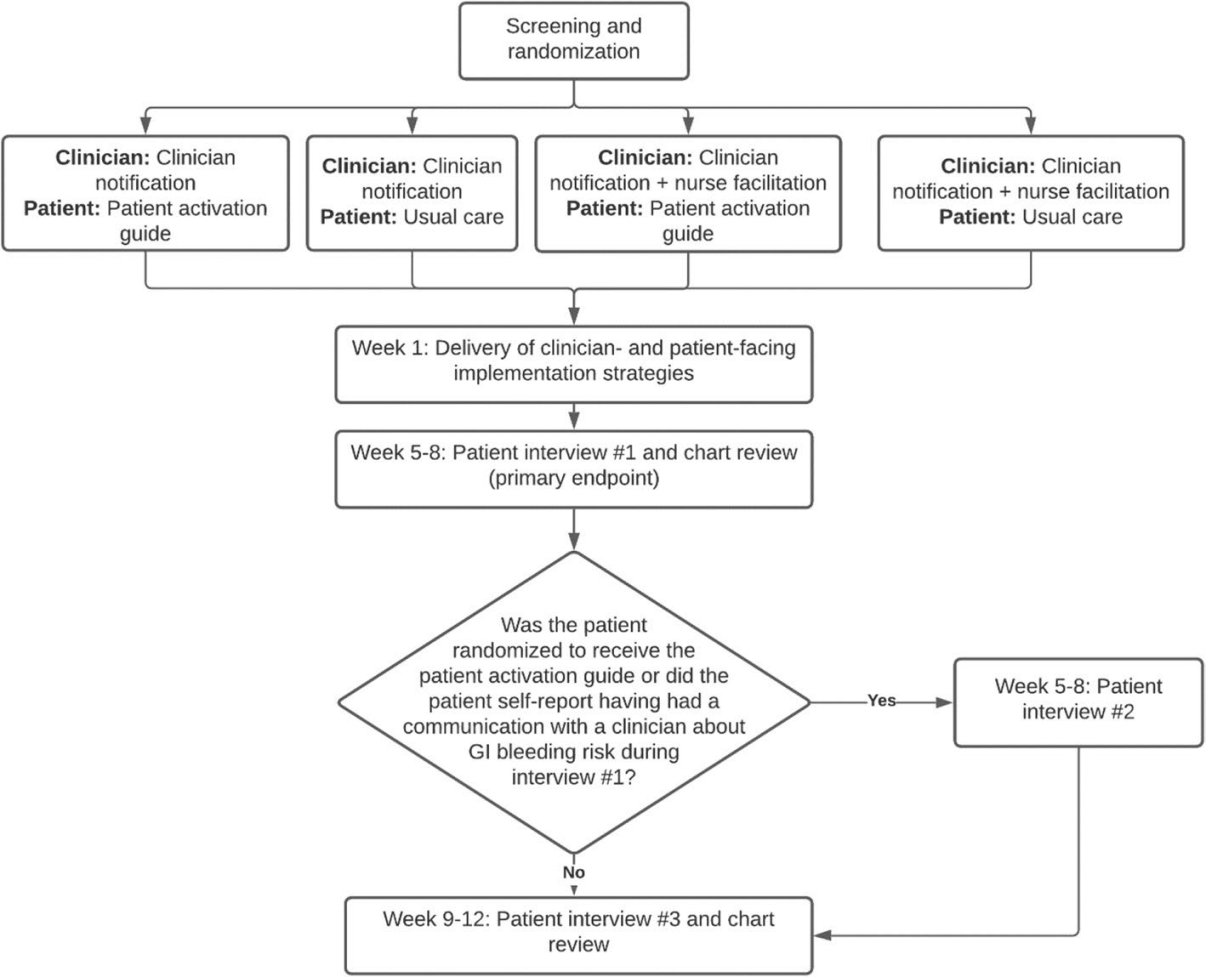
Study flow diagram for patients

The design of the pilot study will be a miniature version of the optimization study that is anticipated as phase 2 of MOST (Fig. 2). This will be a pilot pragmatic cluster- and individual-level randomized controlled factorial implementation trial. A stratified random sample of target clinicians, consisting of 6 clinicians in cardiology and 6 clinicians from other specialties, will be selected for participation. These target clinicians will be randomized 1:1 to receive either CN or CN+NF for all patients for whom they are the designated target clinician. Cluster randomization at the clinician level was employed to avoid having clinicians receive both types of clinician-level intervention, which might have resulted in uncertain expectations of the AMS. Separately, patients will be individually randomized to receive the activation guide or to patient communication as usual. We anticipate 50 patients cared for by the selected target clinicians will be included in this pilot trial.

The pilot trial has a 2x2 factorial design. This design was selected because in a future optimization trial, it will allow us to compare simultaneously the effectiveness of CN vs. CN+NF, and the patient activation guide vs. usual care. However, for the purposes of the pilot study, the primary aims are to (1) evaluate the feasibility of recruiting patients to participate in study assessments as part of a randomized quality improvement trial, (2) evaluate the feasibility of delivering the patient and clinician intervention components as intended, and (3) evaluate multiple additional implementation outcomes for each of the intervention components.

Because this is a pilot intervention study, if challenges with the feasibility of intervention delivery or with study procedures are identified, they may be modified during the trial, with appropriate IRB approval.

#### Delivery of the trial interventions and fidelity

There will not be a formal recruitment process for patient or clinician participants receiving the intervention since the implementation strategies are being delivered with the intent of the quality improvement using a pragmatic approach. Two registered nurses from the AMS, who were members of the participatory planning group, will deliver CN, CN+NF, and the activation guide. One nurse will deliver interventions to the 6 clinicians (and their patients) assigned to receive CN (+/− the activation guide), and the other will deliver interventions to the 6 clinicians (and their patients) assigned to receive CN+NF (+/− the activation guide). In preparation for the trial, both nurses will participate in a 60–90-min training session and practice delivery of the interventions in a practice EHR environment.

#### Quantitative endpoints

The primary endpoint is the proportion of patients able to be contacted and willing to participate in a brief interview between weeks 5–8 (“patient interview 1,” described below) after up to three contact attempts. Patients will be counted as meeting the primary endpoint if they complete the interview. This endpoint will provide crucial information about the feasibility of ascertaining medication changes (i.e., effectiveness) as part of a future trial. The secondary endpoint will be defined as the proportion of patients who received the assigned implementation strategies (inclusive of CN, CN+NF, and the activation guide) during the week of intended delivery and will be determined by EHR chart review at the end of the trial. When any of the implementation strategies is delivered, a timestamp is recorded in the EHR.

We will also evaluate multiple exploratory outcomes (Supplement 11). Data for exploratory endpoints will be gathered through a combination of EHR review at multiple timepoints and structured interviews, described below. These endpoints include the prevalence of warfarin discontinuation during the study period, the accuracy of using the EHR for ascertaining inclusion criteria and for ascertaining changes in medications during the study, the feasibility of collecting complete study data, the feasibility of delivering the clinician- and patient-level interventions, the feasibility of prompting patient-clinician communication about medication optimization using the interventions, various process measures related to use of each of the clinician- and patient-level interventions, the appropriateness of antiplatelet medication use at baseline and at the end of the study, and the effectiveness of the implementation strategies for achieving medication optimization. The last of these is anticipated to be the primary outcome in a future optimization trial.

#### Quantitative analysis

For analysis of the primary endpoint, we will use generalized linear mixed effects modeling (logit link) to estimate the proportion of randomized patients that completed the patient assessment at week 5. This model will include an effect for the individual-level intervention, an effect for the clinician-level intervention, an interaction between the individual and clinician-level intervention and a random effect for clinician. We will report the odds ratios with corresponding confidence intervals for the main individual- and clinician-level intervention effects. A similar model will assess for the secondary endpoint of the proportion of randomized patients that received all implementation components.

Exploratory analyses will mainly consist of descriptive analyses using cross-tabulations, means, standard deviations, and proportions and controlling for clinician type in our mixed models. The accuracy, positive predictive value, and negative predictive value will be calculated for antiplatelet therapy and PPIs at multiple time points, comparing the EHR medication list to self-report (reference standard). The exploratory effectiveness endpoint will be analyzed using an intention-to-treat approach and will account for clustering at the clinician level and repeated measures using mixed effect modeling.

#### Interviews

The research team will conduct interviews with patients, clinicians, and anticoagulation nurses by telephone or Zoom videoconference (Zoom Video Communications, Inc.). Interviews will be recorded with participants’ permission. The contents of these interviews, focused mostly on feasibility and acceptability, are described below. For interviews with patients, clinicians, and anticoagulation nurses, questions related to acceptability were developed based on the seven constructs contained within the Theoretical Framework of Acceptability [53].

##### Interviews with patients

Patient interview 1 is a structured interview that will be conducted between weeks 5–8 and contain questions related to medication use at the time of randomization and at the time of the call, and the patient’s engagement with the activation tool (for patients randomized to receive it). This assessment, which is anticipated to last 5–10 min, was scheduled as such because we hypothesize that most medication optimization will occur within the first month following the interventions.

Patient interview 2 is a semi-structured interview that will be conducted with the subset of patients who were either randomized to receive the activation guide or who in assessment 1 reported that they had communicated with their clinicians about medication optimization. The interview will be scheduled for shortly after patient interview 1. The interview will explore how patients received the activation guide (mail or portal), initial perceptions, acceptability, understanding of the intent, perceived clarity, relevance to their health, whether they read through the guide, and recall of the main message. Images from the activation guide may be shared with patients over Zoom to facilitate recall. The interview will next explore the role that the guide played in any medication changes, specifically whether patients communicated with their clinicians about medication optimization, when it occurred relative to intervention, the mode of communication (portal messages, phone calls, in-person discussion), who initiated the communication, patients’ perceptions of the communication or discussion, including perceived receptiveness of the clinician, and the usefulness of the scripted questions provided in the activation guide. The interview will also explore ways in which the guide might have been improved. We will also administer a validated four-item acceptability measure (Acceptability of Intervention Measure [AIM]) [54].

Patient interview 3 will be a brief structured interview that pertains to current medication use, and 30-day adherence to anti-platelet therapy and/or PPI using a validated three-item self-report for medication adherence [55]. It will occur at weeks 9–12.

##### Interviews with clinicians

All clinicians who participated in the study will be invited to participate in a one-time semi-structured qualitative interview, which will take place at 5–10 weeks after the last patient in the clinician-cluster entered the study. The interview will start with a broad exploration of clinicians’ perceptions of and experiences receiving CN or CN+NF, including any action taken in response to the message, the elapsed time between intervention and medication optimization, and involvement of the patient and/or other clinicians in medical decision making. Next, the interview will cover the acceptability of the implementation strategies, fit with the clinician’s workflow, and features that were particularly well liked or that could have been improved. Clinicians who received CN+NF will be asked about the perceived accuracy of the clinical information provided by the anticoagulation staff and the usefulness of order entry and patient education performed by the anticoagulation clinic staff.

For clinicians who did not recall receiving the notification, the interview will include a description of the intervention, after which the clinician’s hypothetical impressions of the implementation strategies will be sought. The interview will also explore steps that might have increased the likelihood of encountering the message (for example, delivery of the message to a different folder in the EHR inbox). For clinicians who had at least one patient randomized to receive the activation guide, the interview will explore clinicians’ opinions of engaging patients in this way, and any specific experiences with patients who had received the guide. Clinicians will also complete the AIM [54].

##### Interviews with nurses

AMS nurses who delivered the interventions will participate in semi-structured interviews at the conclusion of the trial. The interviews will cover acceptability, feasibility, including the process of extracting relevant clinical information for patients randomized to CN+NF, barriers encountered, perceptions of the informatics tools used, and the amount of time required to deliver the interventions. We will also administer the four-item Intervention Appropriateness Measure, the four-item Feasibility of Intervention Measure, and the AIM [54].

#### Qualitative analysis

Data collected during patient interview 2 as well as the clinician and nurse interviews will be analyzed using a rapid deductive analysis approach guided by the Theoretical Framework of Acceptability (TFA) [53, 56, 57]. We will develop a structured summary template for recording information focusing on acceptability, as defined by the TFA component constructs (affective attitude, burden, perceived effectiveness, ethicality, intervention coherence, opportunity costs, and self-efficacy), barriers to use, and opportunities for enhancement. Immediately after each interview, a primary analyst will use the template to construct an initial interview summary based on interview notes. Preliminary interpretations will also be documented as part of the summary. The primary analyst will then update the summary using either the interview audio recording or transcript, which includes confirming information from the interview notes and adding information as needed along with supporting quotes. For a subset of interviews, a secondary analyst will review the summary after listening to the audio recording or reading the transcript and use comments and colored text to highlight any additions or edits. Discrepancies will be resolved through discussion. These summaries will be discussed by the research team and used to develop matrices that facilitate pattern recognition and interpretation. The information gleaned will be instrumental in identifying issues related to the acceptability of the implementation strategy components and inform any necessary modifications in preparation for testing in a larger trial.

#### Sample size and power

The trial will include 12 target clinicians (6 cardiologists and 6 clinicians in other specialties). In addition, all patients within each clinician cluster will be included. We anticipate that a total of 50 patients will be included, and the nursing staff will deliver the initial interventions to 8 clinician-patient dyads per week. Thus, intervention delivery will be completed over the course of 6–7 consecutive weeks. The feasibility assessment will help confirm that we can undertake a larger factorial trial on the primary endpoint of interest with adequate power. Although we have a small number of clinicians and patients, and the study is not powered to estimate effectiveness, generalized linear mixed effects models should provide reasonably unbiased estimates of the intervention effects with at least 10 clinicians [58].

#### Randomization procedure

For the clinician-level interventions, to achieve balance in randomization and minimize bias, stratified block randomization (within the strata of cardiologists and non-cardiologists) will be used. For the patient-level interventions, patients are randomized 1:1 to each intervention within each clinician. A similar process will be used to randomly assign patients to a week when they will receive their interventions. If a patient who was initially randomized is found to be ineligible at the start of their assigned week, patients from subsequent weeks will be moved up.

#### Data management

Members of the research team will review all source documents in the EHR. All semi-structured interviews will be audio recorded and transcribed with the permission of the participants. All structured data elements will be entered into a secure and HIPAA-compliant REDCap web application database (REDCap, Vanderbilt University) to ensure data integrity. All data from the study will be accessible only by the research team and will otherwise be kept confidential.

#### Human subject protection

The study has been approved the Institutional Review Board at the University of Michigan. A waiver of informed consent was granted for delivery of the patient and clinician interventions, as well as for patient interview 1, because they constitute quality improvement. Informed consent will be obtained for patient interviews 2 and 3, and interviews with clinicians and anticoagulation clinic staff.

## Discussion

Upper GI bleeding is one of the most common serious complications of anticoagulation; however, medication optimization strategies to reduce the risk in this population are underused. This paper describes the development of patient- and clinician-facing implementation strategies to reduce upper GI bleeding risk in patients prescribed anticoagulant-antiplatelet therapy and treated by an AMS, and the design of a pragmatic pilot factorial randomized controlled implementation trial with the central goal of preparing for a larger optimization study powered to test effectiveness. The primary endpoint of the pilot trial will be patient participation in a telephone interview after 5 weeks, without prior recruitment or informed consent, to ascertain medication use, which will inform the feasibility of using this same ascertainment strategy for the primary endpoint in an optimization trial; the secondary endpoint will be receipt of the appropriately timed implementation interventions. We also plan a qualitative analysis of the feasibility and acceptability of the interventions from the perspective of patients, clinicians, and AMS staff, which will allow us to refine the interventions if necessary. Additional exploratory objectives encompass various process measures, effectiveness, and the appropriateness of changes in therapy.

Together with a participatory planning group, we developed the implementation strategies and evaluation plan using the 5-step process of IM, which helped to ensure they systematically address major barriers to the EBPs and are rooted in behavior change theory, primarily SDT [34]. Based on evidence that simple interventions targeting clinicians have had limited effectiveness in this setting, we developed implementation interventions targeting both patients and clinicians. All intervention strategies were designed to be delivered by AMS staff, in keeping with the pragmatic design. For patients, we created and pre-tested an activation guide, which provides knowledge, encouragement, and scripted language to help patients discuss medication optimization with their clinicians. While patient-facing interventions (information mailings) have been included in previous multicomponent interventions to reduce upper GI bleeding risk, their independent effects have not been evaluated [23, 29]. For clinicians, we developed two interventions. In the first (CN), AMS staff will send a templated message through the EHR that identifies patients as high risk for upper GI bleeding, provides a summary of optimization strategies (either PPI initiation or discontinuation of antiplatelet therapy), and a link to guideline summaries on appropriate use of anticoagulant-antiplatelet therapy to help clinicians decide between the two options. In the second, (CN+NF), anticoagulation staff will send a similar templated message, but it will also include information on the patient’s specific indication for antiplatelet therapy and the relevant portion of the antiplatelet guideline summary within the body of the message. The anticoagulation nurse will also offer to facilitate ordering of a PPI, if indicated, and to provide patient education. CN+NF is intended to allow clinicians to seamlessly decide on a plan of care with minimal extra effort or delay.

While IM is typically used to develop a single unified implementation plan, early tasks in the process led us to believe that patient activation and a more active role for AMS nurses had potential to improve use of the EBPs. The pursuit of these hypotheses had substantial implications for the development and evaluation process. For IM steps 2–4, we created multiple sets of implementation outcomes, performance objectives, change objectives, and implementation protocols and materials depending on whether the patient activation guide were incorporated, and whether or not CN vs CN+NF was used. These hypotheses also necessitated an evaluation and optimization framework capable of identifying those intervention components which are active. Our use of the MOST framework led us to design a multilevel factorial trial, with both cluster and individual randomization, suitable for comparing the effectiveness of CN vs. CN+NF and patient activation vs. treatment as usual. The process of using IM and MOST together was not linear, but rather required iterative refinements of both the implementation strategies and the trial design to ensure that the patient- and clinician-facing implementation interventions could be deployed coherently to patients and clinicians in real-world practice. For example, we recognized that if clinicians received both CN and CN+NF messages for different patients under their care, this would likely cause confusion about the nature of the services offered by the AMS. This consideration led us to use cluster randomization so that each clinician only receives a single intervention type. To our knowledge, this is the first description of an intervention development and evaluation process that explicitly combines the use of IM and MOST in this way.

Several other features of the trial are distinctive: (1) The trial takes a clinically nuanced approach to upper GI bleeding risk reduction, prompting clinicians to consider the appropriateness of two EBPs for every patient. We anticipate discontinuation of anti-platelet therapy will be appropriate for most patients, which if accomplished would reduce the risk of hemorrhage from anatomic sites beyond the GI tract. (2) The trial utilizes nurses to perform outreach to clinicians and patients. Prior interventions have mainly used clinical pharmacists [16, 20, 28–30, 59]. Nurse-led interventions aimed at medication safety are likely to be more scalable and cost-effective and could be incorporated into population health management approaches in the future. (3) The implementation plan provides explicit guidance on which clinicians to target (cardiologists preferentially) when there is >1 candidate. Our qualitative research revealed that cardiologists were likely best positioned to decide on the appropriate use of antiplatelet drugs and gastroprotection. This insight gained through qualitative interviews reenforces the importance of comprehensive assessment of barriers and facilitators to use of EBPs during intervention development. (4) Although justified because of the aims, the use of cluster and individual randomization is unusual for a trial such as this.

### Limitations

The trial has several limitations. First, it relies on the accuracy of the EHR medication list for identifying patients who would benefit from medication optimization. The EHR can contain inaccuracies, especially because PPIs and aspirin are available over the counter. However, our needs assessment suggested that the EHR is suitably accurate. We plan to further explore the accuracy of the EHR as one of our exploratory objectives. Second, the trial is focused exclusively on the use of PPIs but not other antisecretory drugs (i.e., histamine-2 receptor antagonists) as a method of gastroprotection. PPIs have the strongest evidence of effectiveness [10, 11]. Third, this trial is focused on patients who use warfarin and antiplatelet drugs since this combination is highly prevalent. Anticoagulated patients with other risk factors (e.g., a history of upper GI bleeding) may benefit from similar interventions in the future [60]. Finally, the trial will take place at a single institution, which will have implications for external validity.

## Conclusions

The findings of this pilot implementation trial will provide valuable information on the feasibility of delivering and evaluating patient- and clinician-facing implementation strategies to reduce upper GI bleeding risk in patients who use anticoagulant-antiplatelet therapy. Once completed, the trial will lay the groundwork for a larger optimization trial.

## Supplementary Information

**Additional file 1: Supplement 1.** STARI Statement R0.docx

**Additional file 2: Supplement 2.** Needs Assessment Patient InterviewsR0.docx

**Additional file 3: Supplement 3.** Needs Assessment - Literature ReviewR0.docx

**Additional file 4: Supplement 4.** Needs Assessment - Med ReconciliationR0.docx

**Additional file 5: Supplement 5.** Matrices of Change ObjectivesR0.docx

**Additional file 6: Supplement 6.** Guideline SummaryR0.docx

**Additional file 7: Supplement 7.** Clinician Letter TemplatesR0.docx

**Additional file 8: Supplement 8.** Patient Activation Guide Pre-Testing R0.docx

**Additional file 9: Supplement 9.** Patient Activation GuideR0.docx

**Additional file 10: Supplement 10.** Anticoagulation Nurse ProtocolsR0.docx

**Additional file 11: Supplement 11.** Exploratory OutcomesR0.docx

## Data Availability

Not applicable.

## Abbreviations

AEGIS: Anticoagulation with Enhanced Gastrointestinal Safety project
AMS: Anticoagulation monitoring service
BCW: Behavior change wheel
CAD: Coronary artery disease
CBSSM: Center for Bioethics & Social Sciences in Medicine
CDC: Centers for Disease Control and Prevention
CI: Confidence interval
CMS: Center for Medicare & Medicaid Services
CN: Clinician notification
CN+NF: Clinician notification + nurse facilitation
EBP: Evidence-based practice
EHR: Electronic health record
ERIC: Expert Recommendations for Implementing Change
FAQ: Frequently asked questions
GI: Gastrointestinal
HBM: Health Beliefs Model
HIPAA: Health Insurance Portability and Accountability Act
I2I: Innovation to Implementation Lab
IM: Implementation mapping
IRB: Institutional review board
MOST: Multiphase optimization strategy
NSAID: Nonsteroidal anti-inflammatory drug
PCI: Percutaneous coronary intervention
PCP: Primary care provider
PPI: Proton pump inhibitor
PPV: Positive predictive value
QI: Quality improvement
RCT: Randomized controlled trial
REDCap: Research Electronic Data Capture
SDT: Self-determination theory
TDF: Theoretical domains framework
UGIB: Upper gastrointestinal bleeding
US: United States
