# Supplementary material for: Development of a multicomponent implementation strategy to reduce upper gastrointestinal bleeding risk in patients using warfarin and antiplatelet therapy, and protocol for a pragmatic multilevel randomized factorial pilot implementation trial
Source: Implement Sci Commun. 2022 Jan 28;3:8. doi: 10.1186/s43058-022-00256-8 (PMC8796614; doi:10.1186/s43058-022-00256-8)
Supplement: Supplementary file 2 — Additional file 2: Supplement 2. Needs Assessment Patient InterviewsR0.docx [file 43058_2022_256_MOESM2_ESM.docx]

# **Supplement 2.** Needs Assessment – Patient Interview Methodology

Interview guides were developed using the theoretical domains framework (TDF)^1^ and iteratively revised to ensure capture of relevant information related to medication optimization to reduce upper GI bleeding risk, with a focus on use of PPI gastroprotection.

We undertook purposive sampling of adult patients who used at least one medication known to increase the risk of upper GI bleeding. Patients were identified and recruited using DataDirect, a software tool that allows identification of potential research participants who meet specific clinical and demographic criteria at Michigan Medicine. Patients were compensated for their participation and received a gift card for $20 if interviewed over the phone, and $30 if interviewed in person. After 5 patient interviews, thematic saturation was achieved.

All interviews were audio-recorded and transcribed verbatim with participants consent. Analytic memos were created for each interview to document the research team’s first impressions within 1-day of interview completion. Each interview transcript was later coded using NVivo software to map quotations to relevant TDF domains and subdomains. This component of the evaluation was approved by the IRB at the University of Michigan. A rapid analysis was performed in which a single research assistant coded excerpts from each transcript to four major themes ^2^.

This component of the evaluation was approved by the IRB at the University of Michigan.

**Table 1. Participant Characteristics**

| **Characteristic** | **N(%)** |
| --- | --- |
| **Gender**  Male  Female | 4 (80%)  1 (20%) |
| **Age**  <60  >60 | 2 (40%)  3 (60%) |
| **Race/ethnicity**  White/Caucasian  Black or African American  Hispanic or Latino/Latina/Latinx | 3 (60%)  1 (20%)  1 (20%) |
| **Marital Status**  Married  Single or never married  Divorced | 1 (20%)  1 (20%)  3 (60%) |
| **Annual Household Income**  <$25,000  $25,000 - $75,000  >$75,000 | 2 (40%)  1 (20%)  2 (40%) |

References

1. Atkins L, Francis J, Islam R, et al. A guide to using the Theoretical Domains Framework of behaviour change to investigate implementation problems. *Implement Sci*. 2017;12(1):77. doi:10.1186/s13012-017-0605-9

2. Gale RC, Wu J, Erhardt T, et al. Comparison of rapid vs in-depth qualitative analytic methods from a process evaluation of academic detailing in the Veterans Health Administration. *Implementation Sci*. 2019;14(1):1-12. doi:10.1186/s13012-019-0853-y
