# Supplementary material for: Development of a multicomponent implementation strategy to reduce upper gastrointestinal bleeding risk in patients using warfarin and antiplatelet therapy, and protocol for a pragmatic multilevel randomized factorial pilot implementation trial
Source: Implement Sci Commun. 2022 Jan 28;3:8. doi: 10.1186/s43058-022-00256-8 (PMC8796614; doi:10.1186/s43058-022-00256-8)
Supplement: Supplementary file 3 — Additional file 3: Supplement 3. Needs Assessment - Literature ReviewR0.docx [file 43058_2022_256_MOESM3_ESM.docx]

# **Supplement 3.** Needs Assessment Literature Review

## Methodology

A rapid literature review was performed to identify any studies describing implementation strategies to improve the use of PPI gastroprotection. The search was conducted in Ovid Medline on May 28, 2020, and used key terms such as gastroprotect*, proton pump inhibitor, intestine*, hemorrhage, implement*, intervention?, and decision making to identify relevant studies on the topic. The search was limited to articles published in English, with no additional filters or limits applied. The full search strategy is available below.

## Search Strategy

((gastroprotect* OR "proton pump inhibitor" or "proton pump inhibitors" OR "proton pumps" OR omeprazole OR prilosec OR esomeprazole OR nexium OR lansoprazole OR prevacid OR rabeprazole OR aciphex OR pantoprazole OR protonix OR dexlansoprazole OR dexilant OR zegerid OR PPI OR (safe* adj5 prescri*)).ab,ti.

AND

((GI or gastrointestinal or ulcer or stomach or duodenal or intestin$) adj5 (bleed$ or risk or h?emorrhage or mel?ena or h?ematemesis)).ab,ti OR (("high risk" or suboptimal) adj5 prescrib$).ab,ti or ("medication error?" or "adverse drug event?" or complication).ab,ti.

AND

(coordinat$ or outreach or communicat$ or "pay for performance" or stakeholder? or ownership or champion? or monitor or "opinion leader" or remind$ or collaborative? or facilitat$ or disseminat$ or "anticoagulation clinic" or "anticoagulation clinics" or alert or alerts or "electronic health record" or “electronic health records” or implement$ or "decision support" or "computer alert" or educat$ or train$ or "decision making" or "decision-making" or pharmac$ or "information technology" or quality or audit? or feedback or safety or incentiv$ or guideline? or support or intervention? or "behavior change" or "behaviour change" or ((patient? or stakeholder) adj5 (involv$ or activat$ or mediat$ or engag$))).ab,ti.)

NOT (intravenous or IV or hospitalized or "stress ulcer" or "critically ill" or "intensive care").ti.)

## Results

The search produced 1,032 results, of which 18 articles describing 15 studies were deemed eligible for inclusion ^1–18^. Forward and backward citation tracking was performed for all eligible articles to identify any additional relevant publications linked to a study. This citation tracking identified the protocol manuscript for the PINCER trial ^19^, but no other relevant publications linked to the included studies. A study team member extracted data from each included study using Microsoft Excel.

While some studies focused solely on improving rates of PPI gastroprotection in a single risk factor group, others included strategies for reducing high-risk prescribing in multiple high-risk patient populations. Of the 15 eligible studies, 14 focused on reducing GI bleeding risk among patients flagged for high-risk NSAID use ^1–6,8–12,14–16^. Only 5 studies included participants at high risk for GI bleeding due to use of combination antithrombotic therapy (CAT) without PPI gastroprotection ^5,6,11,13,16^. Ten studies utilized a medication optimization outcome, in which the study team considered either discontinuation of a high-risk drug or initiation of a gastroprotective drug to be a favorable outcome ^2,5,6,8,9,11–13,15,16^ while another 5 studies looked solely at initiation of a gastroprotective drug ^1,3,4,10,14^. In all studies, prescription of a PPI was considered appropriate for risk reduction. However, all but one study also considered a participant to be at reduced risk for bleeding if they were prescribed another gastroprotective drug, with misoprostol considered appropriate in 10 studies ^2–4,6,8–10,12,15,16^, and 8 studies that considered a single ^4,5,11,13,14,16^ or double dose ^12,15^ of an H2 blocker to be appropriate. One study considered a patient to have received appropriate gastroprotection if they used any of 5 medications, including a PPI, double dose H2 blocker, misoprostol, zinc acexamate or dosmalfate ^12^.

The strategies most widely tested for reducing GI bleeding risk included provider feedback tools such as computerized alerts, dashboards, or clinical decision support systems, which were utilized in 8 studies ^1,2,5,6,8,9,11,16^, and provider education strategies, which were used in 6 studies ^2,4–6,10,11^. Three studies (of which the results of one are unpublished to date ^7^) additionally included direct patient outreach strategies in conjunction with provider-focused interventions ^12,13,16^. However, one of these studies ^13^ only reported on the process for screening high-risk patients and did not publish results on the effect of the patient-facing outreach. In another study ^2^, patient education materials were made available to clinicians, but not directly sent to patients thus requiring mediation of the patient-facing intervention through the provider, placing added responsibility for risk reduction on the healthcare team. Indeed, while previous studies have described strategies for including patient-facing strategies in safety initiatives, rigorous evaluations of the effects of these interventions are needed to assess whether these efforts are beneficial and cost-effective, particularly in settings where clinician and staff time is limited.

Two multi-level intervention studies noted significant improvements in rates of appropriate gastroprotection at 6-months post intervention ^1,7^. However, in both studies, these improvements were no longer significant, and rates had returned to near baseline levels by 12-months post intervention, highlighting the need for sustainment of medication optimization strategies.

## References

1. Avery AJ, Rodgers S, Cantrill JA, et al. A pharmacist-led information technology intervention for medication errors (PINCER): a multicentre, cluster randomised, controlled trial and cost-effectiveness analysis. *Lancet*. 2012;379(9823):1310-1319. doi:10.1016/S0140-6736(11)61817-5

2. Gill JM, Mainous AG 3rd, Koopman RJ, et al. Impact of EHR-based clinical decision support on adherence to guidelines for patients on NSAIDs: a randomized controlled trial. *Ann Fam Med*. 2011;9(1):22-30. doi:10.1370/afm.1172

3. Laine L, Connors L, Griffin MR, Curtis SP, Kaur A, Cannon CP. Prescription rates of protective co-therapy for NSAID users at high GI risk and results of attempts to improve adherence to guidelines. *Aliment Pharmacol Ther*. 2009;30(7):767-774. doi:10.1111/j.1365-2036.2009.04090.x

4. Lanas A, Esplugues JV, Zapardiel J, Sobreviela E. Education-based approach to addressing non-evidence-based practice in preventing NSAID-associated gastrointestinal complications. *World J Gastroenterol*. 2009;15(47):5953-5959. doi:10.3748/wjg.15.5953

5. Dreischulte T, Donnan P, Grant A, Hapca A, McCowan C, Guthrie B. Safer Prescribing--A Trial of Education, Informatics, and Financial Incentives. *N Engl J Med*. 2016;374(11):1053-1064. doi:10.1056/NEJMsa1508955

6. Guthrie B, Kavanagh K, Robertson C, et al. Data feedback and behavioural change intervention to improve primary care prescribing safety (EFIPPS): multicentre, three arm, cluster randomised controlled trial. *BMJ*. 2016;354:i4079. doi:10.1136/bmj.i4079

7. Wallis KA, Elley CR, Lee A, Moyes S, Kerse N. Safer Prescribing and Care for the Elderly (SPACE): Protocol of a Cluster Randomized Controlled Trial in Primary Care. *JMIR Res Protoc*. 2018;7(4):e109. doi:10.2196/resprot.9839

8. Berner ES, Houston TK, Ray MN, et al. Improving ambulatory prescribing safety with a handheld decision support system: a randomized controlled trial. *J Am Med Inform Assoc*. 2006;13(2):171-179.

9. Berner ES, Kasiraman RK, Yu F, Ray MN, Houston TK. Data quality in the outpatient setting: impact on clinical decision support systems. *AMIA Annu Symp Proc*. 2005;(101209213):41-45.

10. Cote GA, Rice JP, Bulsiewicz W, et al. Use of physician education and computer alert to improve targeted use of gastroprotection among NSAID users. *Am J Gastroenterol*. 2008;103(5):1097-1103. doi:10.1111/j.1572-0241.2008.01907.x

11. Grant AM, Guthrie B, Dreischulte T. Developing a complex intervention to improve prescribing safety in primary care: mixed methods feasibility and optimisation pilot study. *BMJ Open*. 2014;4(1):e004153. doi:10.1136/bmjopen-2013-004153

12. Ibanez-Cuevas V, Lopez-Briz E, Guardiola-Chorro MT, NSAID induced Gastropathy Prevention Programme Group. Pharmacist intervention reduces gastropathy risk in patients using NSAIDs. *Pharm World Sci*. 2008;30(6):947-954. doi:10.1007/s11096-008-9258-8

13. Jackson AN, Kogut S. Use of electronic personal health records to identify patients at risk for aspirin-induced gastrointestinal bleeding. *Consult Pharm*. 2013;28(5):313-318. doi:10.4140/TCP.n.2013.313

14. Kim SJ, Han KT, Kang HG, Park EC. Toward safer prescribing: evaluation of a prospective drug utilization review system on inappropriate prescriptions, prescribing patterns, and adverse drug events and related health expenditure in South Korea. *Public Health*. 2018;163(qi7, 0376507):128-136. doi:10.1016/j.puhe.2018.06.009

15. Teichert M, Griens F, Buijs E, Wensing M, De Smet PAGM. Effectiveness of interventions by community pharmacists to reduce risk of gastrointestinal side effects in nonselective nonsteroidal anti-inflammatory drug users. *Pharmacoepidemiol Drug Saf*. 2014;23(4):382-389. doi:10.1002/pds.3587

16. Wallis KA, Elley CR, Moyes S, Kerse N. Safer Prescribing and Care for the Elderly (SPACE): a pilot study in general practice. *BJGP Open*. 2018;2(3):bjgpopen18X101594. doi:10.3399/bjgpopen18X101594

17. Wallis K, Tuckey R. Safer Prescribing and Care for the Elderly (SPACE): feasibility of audit and feedback plus practice mail-out to patients with high-risk prescribing. *J Prim Health Care*. 2017;9(2):145-152. doi:10.1071/HC17018

18. Guthrie B, Treweek S, Petrie D, et al. Protocol for the Effective Feedback to Improve Primary Care Prescribing Safety (EFIPPS) study: a cluster randomised controlled trial using ePrescribing data. *BMJ Open*. 2012;2(6):e002359. doi:10.1136/bmjopen-2012-002359

19. Avery AJ, Rodgers S, Cantrill JA, et al. Protocol for the PINCER trial: a cluster randomised trial comparing the effectiveness of a pharmacist-led IT-based intervention with simple feedback in reducing rates of clinically important errors in medicines management in general practices. *Trials*. 2009;10(1):28. doi:10.1186/1745-6215-10-28
