# Supplementary material for: Development of a multicomponent implementation strategy to reduce upper gastrointestinal bleeding risk in patients using warfarin and antiplatelet therapy, and protocol for a pragmatic multilevel randomized factorial pilot implementation trial
Source: Implement Sci Commun. 2022 Jan 28;3:8. doi: 10.1186/s43058-022-00256-8 (PMC8796614; doi:10.1186/s43058-022-00256-8)
Supplement: Supplementary file 4 — Additional file 4: Supplement 4. Needs Assessment - Med ReconciliationR0.docx [file 43058_2022_256_MOESM4_ESM.docx]

# **Supplement 4**. Anticoagulation Clinic Medication Reconciliation to Assess Accuracy of the Electronic Health Record (EHR)

## Medication Reconciliation Methodology

We undertook a brief evaluation of the accuracy of aspirin and PPI documentation in the EHR medication list for patients followed by the anticoagulation service. A brief questionnaire was developed to ascertain the use of aspirin and PPIs during a brief structured phone survey. Our primary interest was in the positive predictive value (PPV) of EHR-documented aspirin, and among patients using anticoagulant-antiplatelet therapy, the positive and negative predictive value (NPV) of EHR-documented PPIs. We also enquired about use of NSAIDs, although this was a secondary objective. We initially contacted a random sample of 60 patients who had an encounter with the anticoagulation clinic in the prior 4 months, and who were documented as using a PPI with CAT, a sample which included 52 patients documented as using aspirin, six using a thienopyridine, and 2 using aspirin plus a thienopyridine. We then called a random sample of 12 patients documented as using CAT without a PPI, all of whom were documented as using aspirin; we ceased calling patients in the second group after reaching only 12 patients because all 12 confirmed that they were not using a PPI. To reach these 72 patients, initial attempts were made to reach 113 patients.

Based on phone calls with these 72 patients, we estimated that for aspirin, the PPV for aspirin was 87.9% (95% confidence interval 82.2, 91.9), and the NPV was 100% (inestimable CI); for PPIs the PPV was 68.3% (95% confidence interval 62.0, 74.1) and the negative predictive value was 100% (inestimable CI). Two-by-two tables can be found in tables 1-4 below. Confidence intervals were calculated using MedCalc (MedCalc, Ostend, Belgium). Our conclusion from this exercise was that we could use the EHR to identify a sample of patients using anticoagulant-antiplatelet therapy without a PPI (i.e., patients who would benefit from medication optimization) with an acceptable rate of false positives. This component of the needs assessment was deemed exempt from IRB oversight since it was a QI project not meeting the definition of human subjects’ research.

## Medication Reconciliation Call Script

**Introduction**

Hi, my name is _____ and I am calling from the Anticoagulation Clinic at Michigan Medicine. We are doing a safety review to make sure we have accurate information in your record on your use of certain medications and medical history that are relevant to the safe use of warfarin [Note: also called Coumadin]. Would it be OK with you if I ask you a few questions about a few medications, so we can make sure they are accurately documented in your electronic health record? This should only take a couple minutes.

[If they only have a minute: Absolutely, would it be okay if I ask you a single question? -- Jump to PPI]

**Questions**

1. In the past 30 days, have you used any of the following medications, known as proton pump inhibitors or acid blockers, that are typically used for treating heartburn or stomach problems?

a. Omeprazole (Prilosec or Zegerid)

Write in response _______________ days

OTC? Yes/no

b. Esomeprazole (Nexium)

Write in response _______________ days

OTC? Yes/no

c. Lansoprazole (Prevacid)

Write in response _______________ days

OTC? Yes/no

d. Pantoprazole (Protonix)

Write in response _______________ days

OTC? Yes/no

e. Dexlansoprazole (Dexilant)

Write in response _______________ days

OTC? Yes/no

f. Rabeprazole (Aciphex)

Write in response _______________ days

OTC? Yes/no

**IF** yes to any, what condition do you use this medication for?

A. Symptoms

B. Gastroprotection

C. Not sure

D. Other: _________________

2. In the past **30 days**, have you used **aspirin (also known as Bayer, Bufferin, Ecotrin, or St. Joseph)?**

Write in response: _____________ days

[If yes] Do you use baby aspirin (81mg) or a full dose aspirin (325mg)? ___________

3. In the past 30 days, have you used any of the following medications, known as non-steroidal anti-inflammatory drugs or NSAIDs, that are typically used for aches and pains?

a. Ibuprofen (Advil or Motrin)

Write in response _______________ days

OTC? Yes/no

b. Naproxen (Aleve, Anaprox DS, Naprosyn)

Write in response _______________ days

OTC? Yes/no

c. Excedrin

Write in response _______________ days

OTC? Yes/no

4. Has a doctor ever diagnosed you with an ulcer or bleeding from your stomach or upper intestines?

Yes/no

Thanks very much. I’m glad we have these medications up to date in your medical record / We will make sure these drugs are accurately documented in your medical record. Have a nice day.

## Medication Reconciliation – Feedback about PPI Use

Based on additional questions asked during these brief medication reconciliation phone calls with clinic patients, we also learned that among PPI users, 26.8% of PPI users purchased the medication over-the-counter, that 29.3% endorsed an indication of gastroprotection, and 90.2% used the PPI all 30 days in the prior month. We also identified 9 patients (12.5%) who endorsed using ibuprofen or naproxen, 5 of whom used it more than half the days in the prior month, and 4 of whom also used a PPI.

Results from Medication Reconciliation Calls

## **Table 1.** Accuracy of Aspirin Documentation in the Electronic Health Record (EHR)

|  | | Patient self-report of current aspirin use during phone call | |  |
| --- | --- | --- | --- | --- |
|  |  | Yes | No |  |
| Documentation of current aspirin use by patient in MiChart | Yes | 58 | 8 | 66 |
|  | No | 0 | 6* | 6 |
|  | | 0 | 6 |  |

*Six patients who were not using aspirin were using a thienopyridine.

## **Table 2.** Positive Predictive Value, Negative Predictive Value, and Accuracy of Aspirin Documentation in the EHR

| Positive Predictive Value (*) | 87.88% | 82.16% to 91.94% |
| --- | --- | --- |
| Negative Predictive Value (*) | 100.00% |  |
| Accuracy (*) | 88.89% | 79.28% to 95.08% |

## **Table 3.** Accuracy of PPI Documentation in the Electronic Health Record

|  | | Patient self-report of current PPI use during phone call | |  |
| --- | --- | --- | --- | --- |
|  |  | Yes | No |  |
| Documentation of current PPI use by patient in MiChart | Yes | 41 | 19 | 60 |
|  | No | 0 | 12 | 12 |
|  | | 41 | 31 |  |

## **Table 4.** Positive Predictive Value, Negative Predictive Value, and Accuracy of PPI Documentation in the EHR

| Positive Predictive Value (*) | 68.33% | 62.00% to 74.06% |
| --- | --- | --- |
| Negative Predictive Value (*) | 100.00% |  |
| Accuracy (*) | 73.61% | 61.90% to 83.30% |
