# Supplementary material for: Development of a multicomponent implementation strategy to reduce upper gastrointestinal bleeding risk in patients using warfarin and antiplatelet therapy, and protocol for a pragmatic multilevel randomized factorial pilot implementation trial
Source: Implement Sci Commun. 2022 Jan 28;3:8. doi: 10.1186/s43058-022-00256-8 (PMC8796614; doi:10.1186/s43058-022-00256-8)
Supplement: Supplementary file 5 — Additional file 5: Supplement 5. Matrices of Change ObjectivesR0.docx [file 43058_2022_256_MOESM5_ESM.docx]

# **Supplement 5.** Matrices of Change Objectives for Implementation Mapping Step 2

Implementation Mapping Step 2. Identify adoption and implementation outcomes, performance objectives, determinants, and change objectives

**Table 1** lists adoption and implementation outcomes and performance objectives for each outcome

- Performance objectives are the specific steps or sub-behaviors that adopters or implementers must perform to meet overall adoption and implementation outcomes and make clear *who* has to do *what* for the program to be adopted, implemented, and continued.

**Table 2** was used to create matrices of change objectives. Matrices cross performance objectives with personal determinants to produce change objectives.

- Change objectives are discrete changes required in each relevant determinant that will influence achievement of the performance objective and answer the question: what *has to change in this determinant in order to bring about the performance objective?*

## **Table 1.** Adoption, Implementation, and Maintenance Outcomes and Performance Objectives

| **Target: Role** | **Adoption, Implementation, and Maintenance Outcomes** | **Performance Objectives** |
| --- | --- | --- |
| **Adopter(s)** | | |
| Michigan Medicine Anticoagulation Clinic: Adopter | The anticoagulation clinic director and management team decides to adopt the AEGIS program and works with site information technology (IT) staff to incorporate program components into the electronic health record (EHR) system. | 1. Agree to participate in AEGIS 2. Agree to set aside staff time to perform chart review and facilitate communication between relevant stakeholders 3. Agree to participate in evaluation of program goals 4. Identify a program champion 5. Gain support from stakeholders (e.g., nurses) |
| **Implementer(s)** | | |
| Anticoagulation Clinic RNs: Implementers (Clinician Notification and Nurse facilitation) | The anticoagulation clinic RN facilitates bleeding risk reduction through identification of patients at high-risk for UGIB, sending patient activation guide when appropriate, chart review and data abstraction, and communication with relevant stakeholders. | 1. RN runs weekly clinician notification and nurse facilitation report to identify high-risk patients 2. For high risk patients, RN completes chart review per clinic protocol and abstracts information regarding indication for antiplatelet therapy 3. RN generates tailored message to the patient’s clinician to help clinician determine necessity of ongoing antiplatelet therapy and/or appropriateness of initiating a PPI for bleeding prevention 4. Anticoagulation clinic staff enter order for PPI upon request from clinician 5. RN communicates recommendation to patient, provides patient education and documents medication change in MiChart upon request |
| Anticoagulation Clinic RNs: Implementers (Clinician Notification) | The anticoagulation clinic RN identifies patients at high-risk for UGIB, alerts the patient’s clinician of the patient’s risk and recommends that the clinician consider making one of two evidence-based medication changes. | 1. RN receives and reviews weekly clinician notification report to identify patients requiring action 2. RN generates message to the patient’s clinician to help clinician determine necessity of ongoing antiplatelet therapy and/or appropriateness of initiating a PPI for bleeding prevention 3. RN documents plan-of-care from clinician response in MiChart |
| Prescribing Clinicians: Implementers | The prescribing clinician reviews communications regarding patient safety and makes a recommendation for a medication change based on the patient’s indication for antithrombotic therapy and medical history. | 1. The clinician regularly reviews communications and notifications sent by the anticoagulation clinic in the EHR 2. Clinician reviews notification +/- patient’s medical history and determines whether ongoing antiplatelet therapy is appropriate 3. Clinician decides to either discontinue antiplatelet therapy or initiate a PPI for gastroprotection 4. Clinician executes the medication change (if clinician received the clinician notification (CN) intervention) and communicates recommendation to RN and patient 5. Clinician communicates recommendation to anticoagulation clinic RN (if clinician received the clinician notification + nurse facilitation (CN+NF) intervention) |
| Anticoagulation Clinic RNs: Implementers (Patient activation guide) | The anticoagulation clinic RN identifies patients at high-risk for UGIB who are randomized to receive the patient activation intervention and sends the tool to patients. | 1. RN reviews the patient activation guide column in the clinician notification or clinician notification and nurse facilitation weekly report 2. RN determines whether patient was randomized to receive the patient activation guide and sends the guide to eligible patients per clinic protocol |
| Patients: Implementers | Patients who are randomized to receive the patient activation intervention will review the activation guide and contact their healthcare clinician to discuss their GI bleeding risk and options to reduce this risk. | 1. Patient receives, reviews and comprehends materials sent by mail or portal 2. Patient contacts their clinician to discuss appropriate strategies for UGIB risk reduction |
| Patients: Implementers | Patient executes any medication changes recommended by their clinician. | 1. Patient purchases new medications (PPI) if recommended by clinician 2. Patient initiates and adheres to recommended medication change |
| **Maintainer(s)** | | |
| Program Champion: Maintainer | The program champion will ensure that the clinic leadership maintains the AEGIS program as part of the clinic’s standard practice for bleeding risk reduction and safe warfarin use for all patients within the clinic, including both established patients and patients newly opened to the clinic’s service. | 1. Program champion discusses with management and clinic director the continuation of the program after evaluation 2. Work with staff managers and clinic director to ensure at least annual review of UGIB risk reports and use of implementation strategies |

Abbreviations: IT = information technology; AEGIS = anticoagulation with enhanced gastrointestinal safety program; EHR = electronic health record; RN = registered nurse; UGIB = upper gastrointestinal bleeding; PPI = proton pump inhibitor

## **Table 2.** Matrices of Change Objectives

| **Adoption Outcomes** | | | | | |
| --- | --- | --- | --- | --- | --- |
| **Program:** AEGIS  **Adopter:** Anticoagulation clinic leadership  **Behavioral outcome:** Anticoagulation clinic leadership agrees to participate in the AEGIS program to reduce upper GI bleeding risk among patients using combination antithrombotic therapy (CAT) | | | | | |
| **Performance Objectives** | **Determinants** | | | | |
|  | **Knowledge** | | **Optimism (or goals)** | **Environmental Context and Resources** | |
| 1. Agree to participate in AEGIS | - Understand the prevalence of CAT without PPI gastroprotection in each clinic - Understand purpose of the project and seriousness of UGIB | | - Leadership and staff are optimistic that the AEGIS project will improve patient safety - Expect that promoting UGIB risk reduction can reduce patient harm and decrease the number of patients needing to temporarily stop anticoagulation following a bleeding event | - Clinic has resources to devote staff effort to the AEGIS project | |
| 2. Agree to set aside staff time to perform chart review and facilitate communication between relevant stakeholders | - Understand importance of addressing appropriateness of antiplatelet therapy and PPI gastroprotection - Understand need for shared decision-making with patient and their antithrombotic prescribers | | - Clinic leadership see benefit in utilizing clinician and staff time to proactively address UGIB risk | - Clinic has resources to devote staff effort to the AEGIS project - Clinic has ability to build templated messages into the EHR to facilitate communicate between clinic staff and clinicians | |
| 3. Agree to participate in evaluation of program goals | - Express knowledge that continuous effort to evaluate outcomes and improve processes is necessary | | - Clinic leadership believe that the evaluation of the AEGIS program will provide useful insight for ongoing quality improvement efforts | - Clinic has ability to build reports into their EHR system to track the prevalence of CAT without PPI gastroprotection among their patients | |
| 4. Identify a program champion | - Know which clinic staff members are capable of and willing to promote the AEGIS program and provide support to other staff members | | - Clinic leadership believes that the program champion can support other staff and increase uptake of the AEGIS project | - Clinics each have one dedicated staff member who has demonstrated high quality care and leadership skills | |
| 5. Gain support from stakeholders | - Know which stakeholders support is needed to promote and sustain the AEGIS program | | - Clinic leadership feels confident that site-specific stakeholders will see the benefit of and support the AEGIS project | - Leadership supports staff spending time working on this patient safety project | |
| **Implementation Outcomes** | | | | | |
| **Implementer:** Nursing staff (RN) as part of clinician notification and nurse facilitation (CN+NF) intervention  **Behavioral outcome:** Anticoagulation clinic RN gathers and communicates accurate clinical information to clinician, who will perform medication optimization, and communicates recommendations to patients as needed. | | | | | |
| **Performance Objectives** | **Determinants** | | | | |
|  | **Knowledge** | | **Skills** | **Environmental Context and Resources** | |
| 1. RN runs weekly clinician notification and nurse facilitation report to identify high-risk individuals | - Understands the procedure for accessing and reviewing report | | - Demonstrates ability to run workbench report and review on a timely basis | - RN has time to dedicate each week to reviewing the report | |
| 2. For high risk patients, RN completes chart review per clinic protocol and abstracts information regarding indication for antiplatelet therapy | - Knows possible indications for antiplatelet therapy - Knows antiplatelet drugs | | - Demonstrates ability to accurately extract information from the patient’s medical record to facilitate decision-making by the clinician | - RN has the time to review patient charts each week - Relevant information in the medical record is easily accessible and accurate | |
| 3. RN generates tailored message to the patient’s clinician to help clinician determine necessity of ongoing antiplatelet therapy and/or appropriateness of initiating a PPI for bleeding prevention | - Knows how to identify which clinician to send a message to | | - Shows confidence in sending the templated message and adding relevant patient information from the medical record | - Tools and templated messages are available within the EHR to support the RN in sending communications to clinicians | |
| 4. Anticoagulation clinic staff enter order for PPI upon request from clinician |  | | - Demonstrates ability to pend orders according to Michigan Medicine policies | - Clear institutional policies on nurse entry of medication orders - Clinicians clearly communicate treatment recommendations to RN | |
| 5. RN communicates recommendation, provides education to patient and documents medication change in MiChart upon request | - RN is able to answer any questions the patient may have | | - RN shows confidence in communicating recommendation, providing education to the patient, and documenting patient communications in the EHR | - “Frequently asked question” document in anticipation of patient questions. - Sustain trusting relationship between patients and anticoagulation clinic and staff. | |
| **Implementer**: Nursing staff (RN) as part of clinician notification (CN) intervention  **Behavioral outcome:** Anticoagulation clinic RN will communicate patient’s high-risk for UGIB to the patient’s clinician, prompting medication optimization to reduce UGIB risk | | | | | |
| **Performance Objectives** | **Determinants** | | | | |
|  | **Knowledge** | | **Skills** | **Environmental Context and Resources** | |
| 1. RN receives and reviews weekly clinician notification report to identify patients requiring action | - Understands the procedure for accessing and reviewing report | | - Demonstrates ability to run workbench report and review on a timely basis | - RN has time to dedicate each week to reviewing the report | |
| 2. RN generates message to the patient’s clinician to help clinician determine necessity of ongoing antiplatelet therapy and/or appropriateness of initiating a PPI for bleeding prevention | - Knows how to identify which clinician to send a message to - Knows how to document contact attempts for tracking purposes | | - Demonstrates ability to accurately identify which clinician to send a MiChart message to - Demonstrates ability to follow clinic protocol | - Tools and templated messages available within the EHR to support the RN in sending communications to clinicians - Clear protocols available for how to send clinician messages | |
| 3. RN documents plan-of-care from clinician response in MiChart |  | | - Demonstrates ability to accurately document any communications in the patient record | - Clear protocols available for how to document communication | |
| **Implementer:** Prescribing clinician  **Behavioral outcome:** Clinician will review communication from an anticoagulation clinic RN and choose to either discontinue antiplatelet therapy or initiate a PPI to ensure medication optimization for the patient | | | | | |
| **Performance Objectives** | **Determinants** | | | | |
|  | **Knowledge** | | **Professional Role** | **Memory, Attention, and Decision Processes** | |
| 1. The clinician regularly reviews communications and notifications sent to them by the anticoagulation clinic in the EHR | - Understands importance of reviewing and responding to clinical communications related to upper GI bleeding risk reduction in a timely manner | |  | - Clinician has time to review communications sent by anticoagulation clinic | |
| 2. Clinician reviews notification +/- patient’s medical history and determines whether ongoing antiplatelet therapy is appropriate | - Knows of evidence on appropriate duration and indications for antiplatelet therapy for various indications and role of antiplatelet therapy in patients on combination antithrombotic therapy - Knows of risks of combination antithrombotic therapy without gastroprotection | |  | - Clinician has readily available and concise guidance on appropriate use of antiplatelet therapy - Clinician commits time and attention to focus on reviewing appropriateness of ongoing antiplatelet therapy | |
| 3. Clinician decides to either discontinue antiplatelet therapy or initiate a PPI for gastroprotection | - Has knowledge of use of PPIs for UGIB prevention - Is well informed of risks and benefits for both medication optimization strategies | | - Believes he/she/they are the correct clinician on the patient’s care team to make decisions about ongoing antiplatelet therapy or PPI initiation, and if not, reaches out to the specialist they believe is responsible for guidance - Believes UGIB risk reduction among anticoagulated patients is an important goal in their professional role | - Clinician applies best available evidence in making decisions about medication optimization (i.e., not overly concerned about PPI risks) | |
| 4. Clinician executes the medication change (if clinician received the clinician notification (CN) intervention) and communicates recommendation to RN and patient | - Understands need to document recommendation in MiChart and communicate to the anticoagulation clinic for patient safety monitoring | | - Believes clinicians are responsible for ensuring closed-loop communication to avoid medical errors | - Clinician remembers to communicate recommendation to RN and patient and document their decision in the EHR - Clinician updates medication list in MiChart to reflect medication change | |
| 5. Clinician communicates recommendation to anticoagulation clinic RN (if clinician received the clinician notification + nurse facilitation (CN+NF) intervention) | - Understands need to document recommendation in MiChart and communicate to the anticoagulation clinic for patient safety monitoring | | - Believes clinicians are responsible for ensuring closed-loop communication to avoid medical errors | - Clinician remembers to communicate recommendation to RN | |
| **Implementer:** Anticoagulation clinic staff  **Behavioral outcome:** Anticoagulation clinic nurse will send the patient activation guide to patients randomized to receive the intervention according to the clinic protocol. | | | | | |
| **Performance Objectives** | **Determinants** | | | | |
|  | **Knowledge** | | **Skills** | **Environmental Context and Resources** | |
| 1. RN receives and reviews the patient activation guide column in the weekly report to identify patients requiring action | - Understands the procedure and acceptable timeline for reviewing list and sending communications | | - Shows confidence in reviewing and monitoring patient list on a timely basis | - RN has time to dedicate each week to reviewing the patient activation guide column in the weekly report | |
| 2. RN determines whether patient was randomized to receive the patient activation guide and sends the guide to eligible patients per clinic protocol | - Knows how to identify from the list which patients should receive the tool | | - Demonstrates ability to accurately identify which patients should receive the activation tool - Demonstrates ability to follow clinic protocol - Demonstrates ability to identify the appropriate clinician the patient should contact and add the contact information to the patient guide cover letter | - RN has a clear clinic protocol to follow to ensure guide is sent properly - Weekly report clearly shows which patients should receive the tool | |
| **Implementer:** Patient  **Behavioral Outcome:** Patients who are randomized to receive the patient activation intervention will review the activation guide and contact their clinician to discuss their GI bleeding risk and options to reduce this risk. | | | | | |
| **Performance Objectives** | **Determinants** | | | | |
|  | **Knowledge** | | **Skills** | **Environmental Context and Resources** | |
| 1. Patient receives, reviews and comprehends materials sent by mail or portal | - Understands purpose of materials - Understands relevance of materials to themselves | | - Shows confidence in assessing personal risk and creating an action plan to talk with their clinician - Has health literacy to understand relevant personal health information | - Clinic has accurate contact information on file for the patient - Patient has timely access to their mail and to the patient portal - Activation guide is written at an appropriate reading level | |
| 2. Patient contacts their clinician to discuss appropriate strategies for UGIB risk reduction | - Identifies from the tool which clinician to contact and what questions to ask their clinician | | - Shows confidence to initiate a conversation with their clinician based on the structured language provided in the guide | - Patient has resources (phone, computer/internet) to contact their clinician | |
| **Implementer:** Patient  **Behavioral Outcome:** Patient executes any medication changes recommended by their clinician. | | | | | |
| **Performance Objectives** | | **Determinants** | | | |
|  |  | **Beliefs about Consequences** | **Knowledge** | | **Environmental Context and Resources** |
| 1. Patient purchases new medications (PPI) if recommended by clinician | | - Patient believes avoidance of GI bleeding is worth cost of medication | - Patient knows of resources to assist with medication costs (health insurance coverage, prescription discount programs) | | - Patient has means to pay for medication |
| 1. Patient initiates and adheres to recommended medication change | | - Patient believes benefits of medication change outweigh potential risks - Patient trusts that clinician is acting in their best interest | - Patient is aware of and understands intended duration of therapy | |  |
| **Maintenance Outcomes** | | | | | |
| **Program:** AEGIS  **Maintainer:** Anticoagulation clinic leadership and program champion  **Behavioral outcome:** Anticoagulation clinic leadership and program champion work to maintain the AEGIS program components as a part of standard clinic care. | | | | | |
| **Performance Objectives** | **Determinants** | | | | |
|  | **Knowledge** | | **Optimism** | **Environmental Context and Resources** | |
| 1. Program champion discusses with management and clinic director the continuation of the program after evaluation | - Know results of the evaluation of the initial program and can communicate results efficiently - Know how prevalent CAT without PPI gastroprotection is to describe extent of the problem | | - Believe continuation of the AEGIS program will be beneficial and result in continuous improvements in patient safety | - Clinic leadership support dedicating staff effort to maintenance of the AEGIS program | |
| 2. Work with staff managers and clinic director to ensure regular review of UGIB risk reports and use of implementation strategies | - Understand effectiveness of the intervention strategies - Know UGIB is a serious problem for patients using anticoagulation | | - Believe that continuing the program will result in sustained improvements in patient safety | - Health IT supports maintenance of EHR reports and tools used to identify patients at high-risk for UGIB and send communications to patients and clinicians | |

Abbreviations: AEGIS = anticoagulation with enhanced gastrointestinal safety program; GI = gastrointestinal; RN = registered nurse; IT = information technology; UGIB = upper gastrointestinal bleeding; CAT = combination antithrombotic therapy; PPI = proton pump inhibitor; CN = clinician notification; CN+NF = clinician notification + nurse facilitation; EHR = electronic health record
