# Supplementary material for: Development of a multicomponent implementation strategy to reduce upper gastrointestinal bleeding risk in patients using warfarin and antiplatelet therapy, and protocol for a pragmatic multilevel randomized factorial pilot implementation trial
Source: Implement Sci Commun. 2022 Jan 28;3:8. doi: 10.1186/s43058-022-00256-8 (PMC8796614; doi:10.1186/s43058-022-00256-8)
Supplement: Supplementary file 6 — Additional file 6: Supplement 6. Guideline SummaryR0.docx [file 43058_2022_256_MOESM6_ESM.docx]

# **Supplement 6.** Guideline Summary on Appropriate Use of CAT

Recommended duration of antiplatelet therapy for patients using anticoagulation, by indication. Clinicians should use their judgment in applying these recommendations to patients depending on the specific clinical scenario.

| **Indication for antiplatelet drug** | | **Recommended management of antiplatelet drug** | **Notes** | **Ref.** |
| --- | --- | --- | --- | --- |
| Primary prevention of coronary artery disease | | | | |
| Primary prevention | | Stop antiplatelet drug |  | ^1^ |
| Treatment of coronary artery disease with atrial fibrillation (AF) | | | | |
| PCI for stable CAD | PCI ≤ 6 months ago | Continue antiplatelet drug | -Clopidogrel preferred  -Consider switch to aspirin 81mg | ^1^ |
|  | PCI >12 months ago | Stop antiplatelet drug |  |  |
| CABG for stable CAD | CABG ≤12 months ago | Continue aspirin 81mg |  |  |
|  | CABG >12 months ago | Stop aspirin |  |  |
| Acute Coronary Syndrome (ACS) +/- PCI | ACS +/- PCI ≤12 months ago | Continue antiplatelet drug | Clopidogrel preferred |  |
|  | ACS +/- PCI >12 months ago | Stop antiplatelet drug |  |  |
| Treatment of coronary artery disease with venous thromboembolism (VTE) | | | | |
| PCI for stable CAD | PCI <6 months ago | Continue antiplatelet drug | -Clopidogrel preferred  -Consider stopping anticoagulant at 3 months if reversibly provoking risk factors | ^1^ |
|  | PCI >6 months ago | Continue antiplatelet drug | -Consider switch to aspirin 81mg  -Consider stopping anticoagulant if reversibly provoking risk factors |  |
|  | PCI >12 months ago | Stop antiplatelet drug | -Consider stopping anticoagulant if reversibly provoking risk factors |  |
| CABG for stable CAD | CABG ≤12 months ago | Continue aspirin 81mg |  |  |
|  | CABG >12 months ago | Stop aspirin |  |  |
| Acute coronary syndrome (ACS) +/- PCI | ACS +/- PCI <3 months ago | Continue antiplatelet drug | Clopidogrel preferred |  |
|  | ACS +/- PCI 3-12 months ago | Continue antiplatelet drug | -Consider switch to aspirin 81mg  -Consider stopping anticoagulant if reversibly provoking risk factors |  |
|  | ACS +/- PCI >12 months ago | Stop antiplatelet drug | -Consider stopping anticoagulant if reversibly provoking risk factors |  |
| Cerebrovascular disease | | | | |
| History of TIA, CVA, or CEA | | Stop antiplatelet drug |  | ^1^ |
| Carotid stent ≤ 3 months ago | | Continue antiplatelet drug | Clopidogrel preferred |  |
| Carotid stent > 3 months ago | | Stop antiplatelet drug |  |  |
| Peripheral arterial disease | | | | |
| Endovascular intervention or surgical repair ≤ 1-3 months ago | | Continue antiplatelet drug | Clopidogrel preferred | ^1^ |
| Endovascular intervention or surgical repair > 1-3 months ago | | Stop antiplatelet drug |  |  |
| Valve replacement | | | | |
| Mechanical Heart Valve | | Stop antiplatelet drug unless another indication is present |  | ^2^ |
| Bioprosthetic Heart Valve ≤ 3 months ago | | Continue aspirin 81mg only if high thromboembolic risk |  |  |
| Bioprosthetic Heart Valve > 3 months ago | | Stop aspirin |  |  |
| TAVR ≤ 3 months | | Continue antiplatelet drug only if high thromboembolic risk |  | ^3^ |
| Venous Intervention (including IVC and Iliofemoral venoplasty/stenting) | | | | |
| Venous procedure ≤2 months prior | | Continue dual antiplatelet therapy |  |  |
| Venous procedure >2 months prior | | -Stop P2Y12 inhibitor  -Continue aspirin 81mg indefinitely |  |  |

PCI=Percutaneous coronary intervention.

**References**

1. Kumbhani DJ, Cannon CP, Beavers CJ, et al. 2020 ACC Expert Consensus Decision Pathway for Anticoagulant and Antiplatelet Therapy in Patients With Atrial Fibrillation or Venous Thromboembolism Undergoing Percutaneous Coronary Intervention or With Atherosclerotic Cardiovascular Disease: A Report of the American College of Cardiology Solution Set Oversight Committee. *Journal of the American College of Cardiology*. 2021;77(5):629-658. doi:10.1016/j.jacc.2020.09.011

2. Writing Committee Members, Otto CM, Nishimura RA, et al. 2020 ACC/AHA Guideline for the Management of Patients With Valvular Heart Disease: A Report of the American College of Cardiology/American Heart Association Joint Committee on Clinical Practice Guidelines. *J Am Coll Cardiol*. 2021;77(4):e25-e197. doi:10.1016/j.jacc.2020.11.018

3. Saito Y, Nazif T, Baumbach A, et al. Adjunctive Antithrombotic Therapy for Patients With Aortic Stenosis Undergoing Transcatheter Aortic Valve Replacement. *JAMA Cardiol*. 2020;5(1):92-101. doi:10.1001/jamacardio.2019.4367
