# Supplementary material for: Development of a multicomponent implementation strategy to reduce upper gastrointestinal bleeding risk in patients using warfarin and antiplatelet therapy, and protocol for a pragmatic multilevel randomized factorial pilot implementation trial
Source: Implement Sci Commun. 2022 Jan 28;3:8. doi: 10.1186/s43058-022-00256-8 (PMC8796614; doi:10.1186/s43058-022-00256-8)
Supplement: Supplementary file 7 — Additional file 7: Supplement 7. Clinician Letter TemplatesR0.docx [file 43058_2022_256_MOESM7_ESM.docx]

# **Supplement 7.** Clinician Notification Letters

## Letter for Clinician Notification Quality Improvement Strategy

[Date]

Dear [clinician name],

As part of an anticoagulation clinic safety initiative, I am contacting you about your patient [patient name] (MRN: [MRN]). This patient is at high risk for upper gastrointestinal bleeding due to combination warfarin and antiplatelet therapy without proton pump inhibitor (PPI) gastroprotection.

Antiplatelet Medications:

[List antiplatelet drugs in patient’s current medication list]

The risk for upper GI bleeding with combination warfarin and antiplatelet therapy is as high as 2.8% per year ^1^. For patients who require ongoing combination warfarin and antiplatelet therapy, professional guidance recommends the use of a proton pump inhibitor ^2^.

Please consider **one** of the following medication changes:

1. **Discontinue antiplatelet therapy if appropriate and remove the antiplatelet drug from the medication list.**

OR

1. **If you determine antiplatelet therapy should continue, prescribe a daily proton pump inhibitor (e.g., omeprazole 20 mg daily) for the duration of combination antithrombotic treatment.** In the prescription, please denote the indication as gastroprotection.

To help with this decision, you may wish to visit this site: Guidance on appropriate use of CAT. [links to full guideline summary available in supplement 6] on the Michigan Medicine intranet, where you can review a concise summary of the recommended duration of combination antithrombotic therapy for various indications.

Please respond to this message by selecting one of the following responses below to alert the anticoagulation clinic service of your plan to address this safety concern.

- I plan to discontinue antiplatelet therapy for this patient.
- I plan to initiate a proton pump inhibitor for the duration of combination antithrombotic therapy.

**Dotphrases you may find useful:**

**.stopantiplt** – Instructions you can forward to your support staff to recommend that the patient discontinue anti-platelet therapy.

**.startppi** – Instructions you can forward to your support staff to recommend that the patient initiate a PPI.

**.antiplateletinquiry** – A message to facilitate discussion of the appropriateness of antiplatelet therapy with a colleague.

Thank you for your time and effort in this important initiative.

Sincerely,

[RN name]

**(Please route back to sender)**

**References:**

1. Scally B, Emberson JR, Spata E, et al. Effects of gastroprotectant drugs for the prevention and treatment of peptic ulcer disease and its complications: a meta-analysis of randomised trials. *Lancet Gastroenterol Hepatol*. 2018;3(4):231-241. doi:10.1016/S2468-1253(18)30037-2

2. Bhatt DL, Scheiman J, Abraham NS, et al. ACCF/ACG/AHA 2008 expert consensus document on reducing the gastrointestinal risks of antiplatelet therapy and NSAID use: a report of the American College of Cardiology Foundation Task Force on Clinical Expert Consensus Documents. *J Am Coll Cardiol*. 2008;52(18):1502-1517. doi:10.1016/j.jacc.2008.08.002

## Letter for Clinician Notification + Nurse Facilitation Quality Improvement Strategy

[Date]

Dear [clinician name],

As part of an anticoagulation clinic safety initiative, I am contacting you about your patient [patient name] (MRN: [MRN]). This patient is at high risk for upper gastrointestinal bleeding due to combination warfarin and antiplatelet therapy without proton pump inhibitor (PPI) gastroprotection.

Antiplatelet Medications:

[List antiplatelet drugs in patient’s current medication list]

The risk for upper GI bleeding with combination warfarin and antiplatelet therapy is as high as 2.8% per year ^1^. For patients who require ongoing combination warfarin and antiplatelet therapy, professional guidance recommends the use of a proton pump inhibitor ^2^.

Please consider **one** of the following medication changes:

1. **Discontinue antiplatelet therapy if appropriate.**

OR

1. **If you determine antiplatelet therapy should continue, prescribe a daily proton pump inhibitor (e.g, omeprazole 20mg daily) for the duration of combination antithrombotic treatment.**

We are providing the following information to help with your decision making. Based on our chart review, the patient uses [antiplatelet drug] for [indication for antiplatelet therapy: (1) primary prevention of CAD, (2) treatment of CAD with atrial fibrillation, (3) treatment of CAD with venous thromboembolism, (4) cerebrovascular disease, (5) peripheral artery disease, (6) valve replacement, (7) venous intervention (including IVC and iliofemoral venoplasty/stenting)].

[Insert only applicable section of guidance summary table from supplement 6 that corresponds with the patient’s identified indication for antiplatelet therapy]

To help with this decision, you may wish to visit this site: Guidance on appropriate use of CAT. [links to full guideline summary available in supplement 6]

If you could please respond to this message and indicate your plan of care, I can communicate the recommendation, along with appropriate education, to the patient, and pend any medication orders for your approval in MiChart. Please cut and paste one of these options in your response to this letter.

- Please communicate to the patient that I would like them to discontinue antiplatelet therapy
- Please communicate to the patient that I would like them to initiate omeprazole 20 mg daily, 90-day supply with 3 refills
- Please communicate to the patient that I would like them to initiate pantoprazole 40 mg daily, 90-day supply with 3 refills

Thank you for your time and effort in this important initiative.

Sincerely,

[RN name]

**(Please route back to sender)**

**References**

1. Scally B, Emberson JR, Spata E, et al. Effects of gastroprotectant drugs for the prevention and treatment of peptic ulcer disease and its complications: a meta-analysis of randomised trials. *Lancet Gastroenterol Hepatol*. 2018;3(4):231-241. doi:10.1016/S2468-1253(18)30037-2

2. Bhatt DL, Scheiman J, Abraham NS, et al. ACCF/ACG/AHA 2008 expert consensus document on reducing the gastrointestinal risks of antiplatelet therapy and NSAID use: a report of the American College of Cardiology Foundation Task Force on Clinical Expert Consensus Documents. *J Am Coll Cardiol*. 2008;52(18):1502-1517. doi:10.1016/j.jacc.2008.08.002
