# Supplementary material for: Development of a multicomponent implementation strategy to reduce upper gastrointestinal bleeding risk in patients using warfarin and antiplatelet therapy, and protocol for a pragmatic multilevel randomized factorial pilot implementation trial
Source: Implement Sci Commun. 2022 Jan 28;3:8. doi: 10.1186/s43058-022-00256-8 (PMC8796614; doi:10.1186/s43058-022-00256-8)
Supplement: Supplementary file 8 — Additional file 8: Supplement 8. Patient Activation Guide Pre-Testing R0.docx [file 43058_2022_256_MOESM8_ESM.docx]

# **Supplement 8.** Patient Activation Guide Pre-Testing Process and Interview Guide

Patient Activation Guide Pre-Testing Process

Patients were either mailed or emailed the activation guide and personalized cover letter and asked to go through the materials at their own pace while saying aloud any thoughts they had about the guide. After a first run-through of the materials, the interviewers then asked participants a series of questions about clarity and acceptability of the activation guide and cover letter. Pre-testing interviews were continued to the point at which further interviews elicited little to no novel information on issues with the activation guide.

Patient Activation Guide Pre-Testing Interview Guide

**Introduction**

Hi, [participant name]. Thank you for your willingness to be interviewed today. My name is [NAME], and I will be leading you through today’s interview.

Before we begin, I have some information for you and I’m going to read through it now to make sure that I cover everything, and you have the chance to ask any questions you might have before we get started.

The purpose of today’s interview is to test materials that will be used by the anticoagulation clinic at Michigan Medicine. These materials were developed specifically for patients who are using warfarin or Coumadin along with a second blood-thinning medication, such as aspirin or Plavix. You may not be using these exact medications. However, your participation in this interview will help us to ensure that these materials are appropriate and understandable for patients who may be able to improve their medication safety. During this interview, we want to be clear that we are testing these materials, and we are not testing you. You can’t do anything wrong here. We encourage you to be as honest as possible and promise that you will not hurt our feelings in any way during today’s interview. Your honest responses will help us make sure that the materials you see today are as useful as possible. As we go through the materials that we have sent you, I am going to ask you to think out loud as much as possible: to say what you’re looking at, what you are thinking, and how anything in the materials makes you feel. This will be a big help to us.

This interview should last under an hour. We understand that your time is very valuable and appreciate you sharing it with us. We would like to get your permission to conduct this interview and video record it in order to facilitate transcription. During the interview you can ask that the recorder be turned off, decline to answer any questions, or stop the interview at any time. All recordings, notes, and transcripts from this interview will be kept confidential and will only be accessible to project staff. After the interview is transcribed, we will remove any information that would allow you to be identified, and the original recording will be destroyed.

Do you have any questions for me about the study, this interview, or the consent process before we begin?

*****Start Recorder*****

And just so that we have it on record, do we have your permission to conduct and video-record this interview?

[If yes, continue to interview guide]

[If no, end interview and thank for their time]

**Interview Questions**

1. Okay, before we look at the materials we sent you, I’d like to ask you just a few quick questions.
   1. First, tell us a little about yourself
      1. **Follow up friendly question**
   2. **Are you retired, or still working?**
      1. **What do/did you do?**
   3. What do you like to do for fun?
   4. Where do you get most of your healthcare?

Great, thank you. Now I would like to move on to the next part of this interview. Please go ahead and open the pdf with the materials that were sent to you for this study.

**Envelope and Cover Letter Questions**

For these next few questions, imagine that you received these materials from the medical center where you receive most of your healthcare either by mail or in your patient portal.

1. From this perspective, looking at the letter that came with the brochure, can you tell us what stands out to you?
   1. What do you notice first?
   2. What strikes you about it?
   3. How do you feel about the information on this page?

Great, now please go ahead and look at the brochure.

1. Looking at the cover of the brochure, please tell me what you are thinking.
   1. What do you notice first?
   2. What strikes you about it?
   3. Where do you think this brochure is coming from?
      1. What makes you think this?
   4. What do you suspect the brochure is for?
      1. Why is that?
   5. How interested would you be to see what’s inside the brochure?
2. If you received this brochure in the mail or your patient portal, what would you do next?
   1. Would you read it right away, or save it for later, or throw it away?

**Brochure First Pass**

Now please read through the brochure just as you would if you were reading this on your own.

Please go through it at the same speed and in the same detail as you might if I wasn’t here. As you’re doing this, please narrate for us what you’re doing. For example, what page you’re looking at, and any first impressions you have.

Probing questions along the way:

1. What are you thinking?
2. What are you looking at?
3. How does this make you feel?
4. Was there something in particular that made you think that?

*If participant has questions or uncertainty:*

- I’d like you to do what you’d normally do if you received this and I was not here.
- This is exactly what we need.
- What do you think? (In response to questions.)

**Brochure Page by Page Review**

**Page 2 – Blood Thinning Medications**

1. Looking at page 2, please tell me what you are thinking.
   1. What do you notice first?
2. How do you feel about the information on this page?
   1. What seems most important?

**Page 3 – Frequently Asked Questions about GI Bleeding**

1. Looking at page 3, tell me what you are thinking.
   1. What do you notice first?
2. Are there any terms on this page you do not know?
3. How do you feel about the information on this page?
   1. What information seems most important?

**Page 4 – Two Options**

1. Looking at page 4, please tell me what you are thinking.
   1. What do you notice first?
2. From your understanding of this page, what can you do to lower your risk of bleeding?
   1. What do you think about these options?
      1. Difficult? Easy?
      2. Are these things you think you would be comfortable discussing with your doctor?

**Page 5 – Proton Pump Inhibitors**

1. Looking at page 5, please tell me what you are thinking.
   1. What do you notice first?
2. How do you feel about the information on this page?
   1. What seems most important?
3. Based on the information on this page, how would you feel if your doctor recommended that you start a PPI to help prevent bleeding?

**Page 6 – Mr. Smith’s Story**

1. Looking at page 6, please tell me what you are thinking.
   1. What do you notice first?
2. How does Mr. Smith’s story make you feel?
3. How realistic does this story seem to you? How much can you relate to this story?

**Page 7 – Mrs. Parker’s Story**

1. Looking at page 7, please tell me what you are thinking.
   1. What do you notice first?
2. How does Mrs. Parker’s story make you feel?
3. How realistic does this story seem to you? How much can you relate to this story?
4. Would you prefer to see these stories at the end of the brochure or at the beginning?

**Page 8 – Questions to Ask Doctor**

1. Looking at page 8, please tell me what you are thinking.
   1. What do you notice first?
   2. What strikes you about it?
2. How do you feel about the questions listed on the page?
3. How comfortable would you feel asking your doctor these questions?
   1. [If no] why?
      1. What *would* you be comfortable asking?
4. Are there any terms on this page you do not know or understand?
   1. Would including a definition for this term on the page be helpful?

ii. Is there a better term we could use?

**Closing Questions**

1. After reading through the whole brochure, which pages did you find to be the most important or most educational?
   1. Why?
   2. Least important or educational?
2. After reading through the full brochure, how do you feel about the length of the brochure?
   1. If we were to make the brochure shorter, which pages do you think are best to remove?
3. If you were a patient reading this, what, if anything, would you do after reviewing the brochure?
   1. [If responds that they would contact their doctor, probe:]
      1. When would you contact them?
      2. Which doctor would you contact?
         1. Can you identify a specific healthcare provider who you would want to contact after reading this?
      3. How would you contact this person?
         1. By phone?
            1. Do you have contact information for that person, or do you know how to find it?
         2. Portal message?
         3. At next appointment?
            1. [If at next appointment] – when is next appointment scheduled for?
   2. **Great, now I would like you to pretend that you received this in the mail, and you are contacting your provider. Please tell me what you would {say to the person who answered the phone at your doctor’s office} or {what your portal message would say exactly}.**
4. After reading through the brochure and cover letter, how would you feel about these materials if you received them in the mail from your healthcare provider?
   1. Would you trust the content?
      1. Why?
      2. What might increase/decrease your level of trust?
5. How much of the information that was presented in the brochure did you already know?
   1. What information was new to you?
6. After reading the brochure, how would you feel about your personal risk of gastrointestinal bleeding?
   1. How concerned are you about having a bleeding event?
      1. On a scale of 1-10?
   2. How much of a priority is it to lower your bleeding risk?
      1. On a scale of 1-10?
7. How much would you want to know about your increased risk for bleeding?
   1. Percentage change in risk?
8. Is there anything you think we could do differently to improve these materials?
9. Are there any changes to the brochure that would make you more likely to contact your doctor about making the medication changes discussed in the brochure?
10. Is there another way the information in this brochure could be communicated that would be preferable to you?
    1. Video
    2. Website link
11. Is there anyone, like a spouse or friend, that helps you with your medical care?
    1. Scheduling appointments
    2. Making medical decisions
    3. Keeping track of medications
12. Do you have a background in healthcare at all?
13. In the screening questions for this study, you stated that you use the following medications: ____________ & _____________.
    1. Can you tell us why you use these medications?
    2. Who prescribes these medications to you?
       1. Cardiologist/Vascular surgeon/PCP/other?
    3. How long have you been using these medications?
14. Before we end this interview, are there any questions that we did not ask that you think would be important to cover in future interviews?

**Thank you very much for taking the time to participate in this interview. We greatly appreciate you sharing your thoughts and insights with us. Do you have any questions for us before we wrap up?**

**As a thank you for taking the time to complete this interview, we will mail you a $20 gift card that can be used anywhere MasterCard is accepted. What is the best address for us to send this to?**
