# Supplementary figures and images for: Development of a multicomponent implementation strategy to reduce upper gastrointestinal bleeding risk in patients using warfarin and antiplatelet therapy, and protocol for a pragmatic multilevel randomized factorial pilot implementation trial
Source: Implement Sci Commun. 2022 Jan 28;3:8. doi: 10.1186/s43058-022-00256-8 (PMC8796614; doi:10.1186/s43058-022-00256-8)

# **Supplement 9.** Final Version of Patient Activation Guide


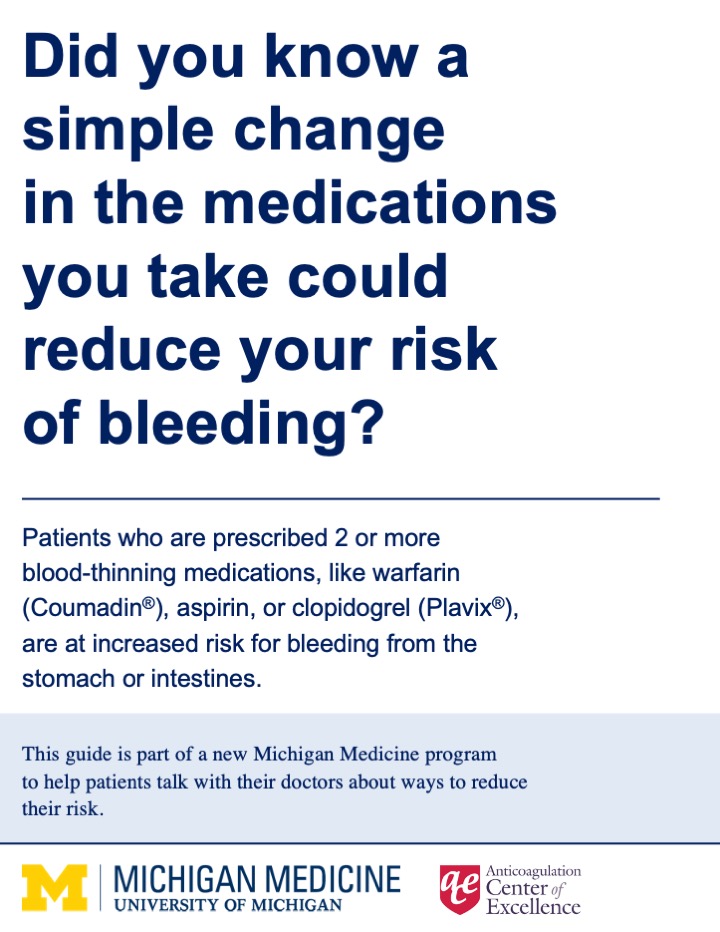


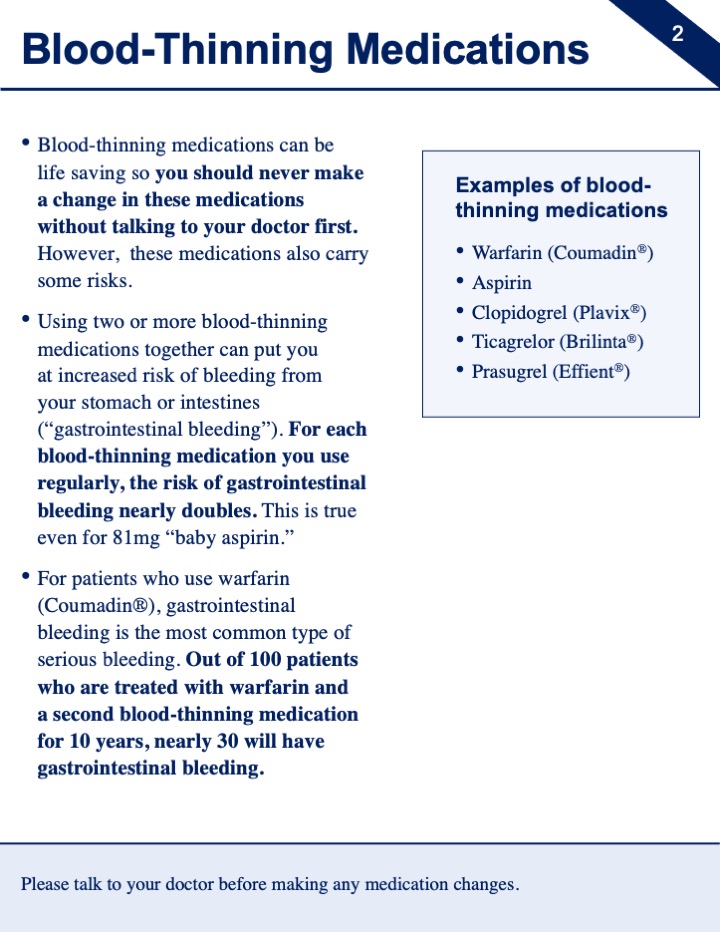


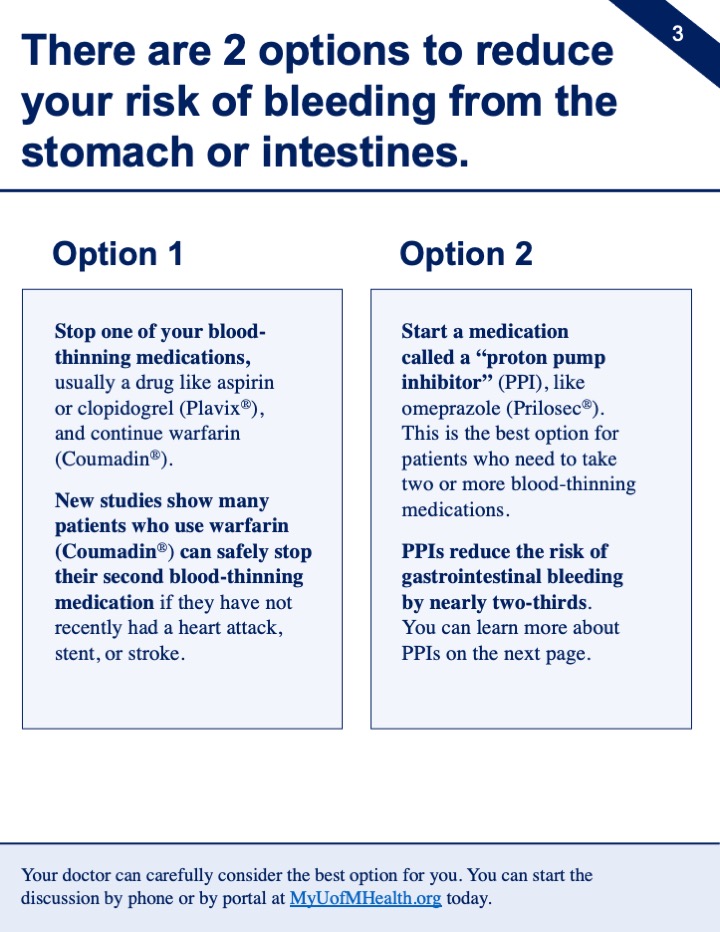


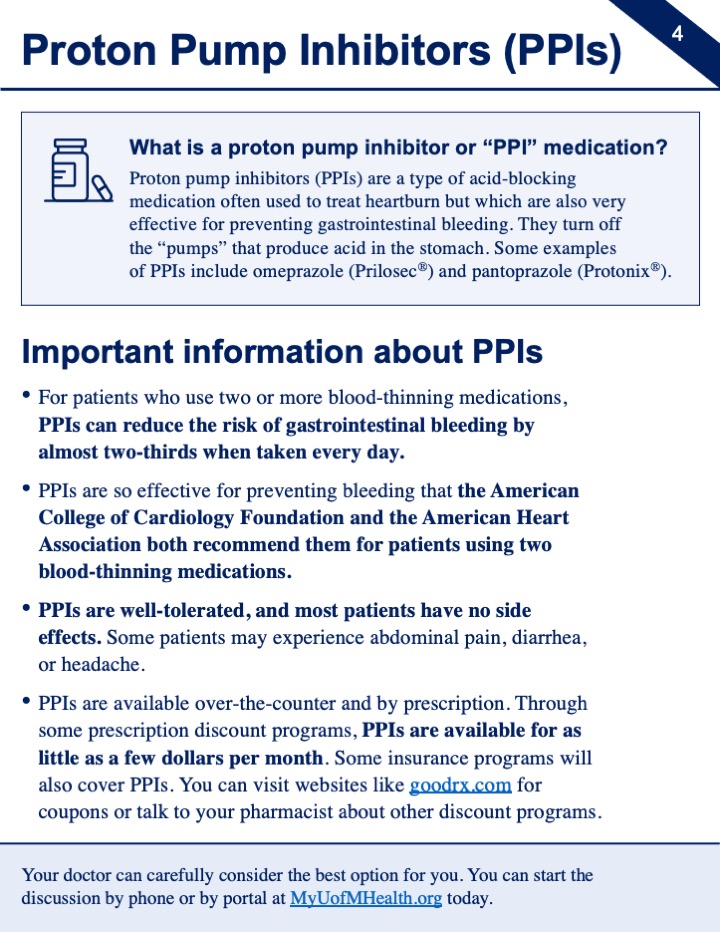


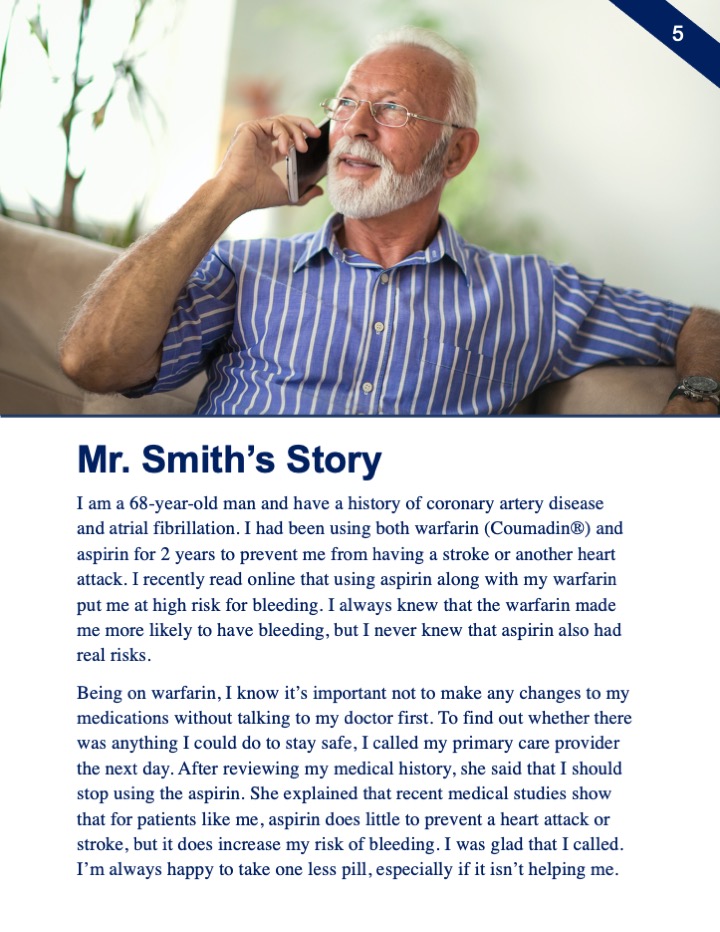


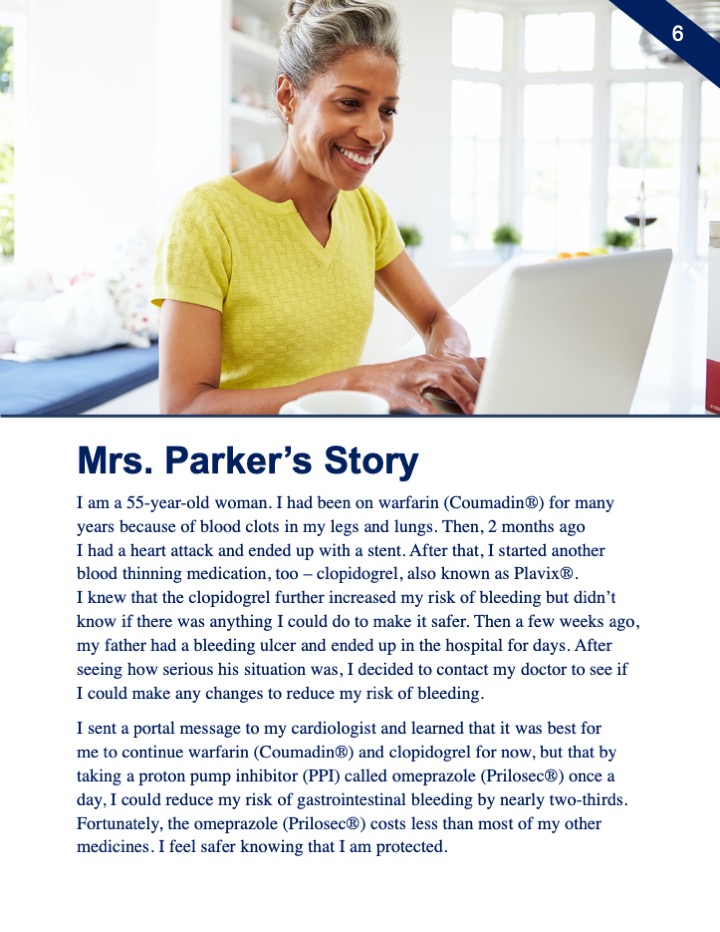


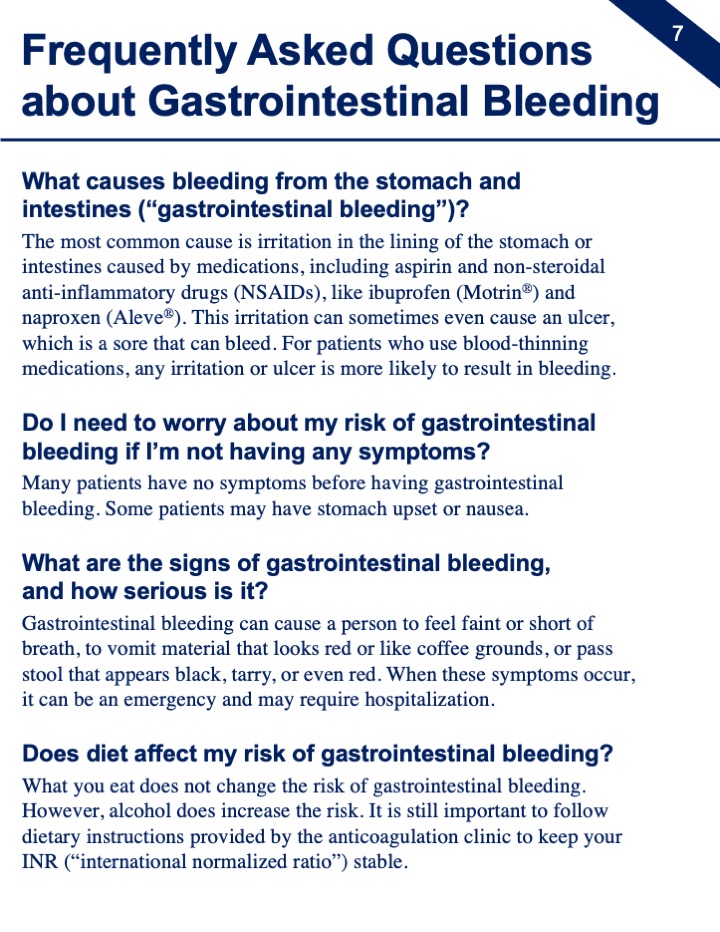


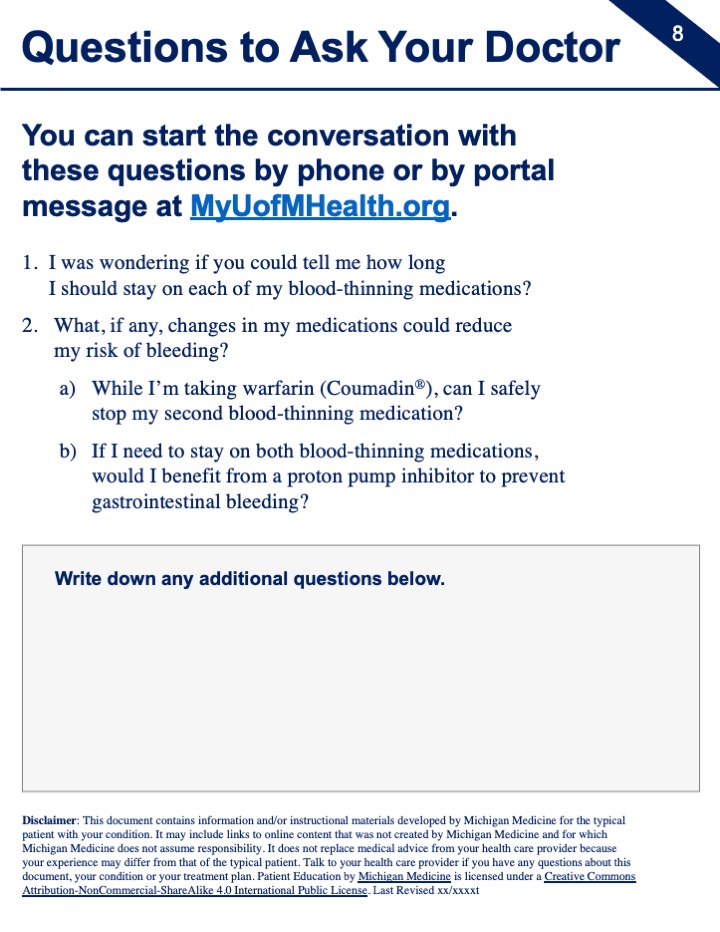

Supplement: Supplementary file 9 — Additional file 9: Supplement 9. Patient Activation GuideR0.docx [file 43058_2022_256_MOESM9_ESM.docx]
