# Supplementary material for: Development of a multicomponent implementation strategy to reduce upper gastrointestinal bleeding risk in patients using warfarin and antiplatelet therapy, and protocol for a pragmatic multilevel randomized factorial pilot implementation trial
Source: Implement Sci Commun. 2022 Jan 28;3:8. doi: 10.1186/s43058-022-00256-8 (PMC8796614; doi:10.1186/s43058-022-00256-8)

# **Supplement 10.** Anticoagulation Clinic Staff Protocols

## Clinician Notification Clinic Staff Protocol for RNs

**Anticoagulation Service**

**Protocol for Upper GI Bleeding Prevention Project**

**Clinician Notification Intervention**

1. **DEFINITION:**

Patients are designated as high risk for upper gastrointestinal (GI) bleeding if they use warfarin along with aspirin (either 325mg or 81mg) and/or a thienopyridine (clopidogrel, prasugrel, ticagrelor) without a proton pump inhibitor (PPI).

1. **PURPOSE:**

This is a quality improvement initiative to help clinicians take appropriate steps to reduce upper GI bleeding risk for patients on warfarin and an antiplatelet drug. As part of this project, a new patient education guide is being trialed. This protocol defines the role of the anticoagulation clinic nurse in this project.

1. **PATIENT ELIGIBILITY:**

All Michigan Medicine Anticoagulation Service patients who are high risk for upper GI bleeding as defined above. Patients with an LVAD, heart transplant, or allergy/intolerance to PPI will be excluded.

1. **PROCEDURE:**

The following steps should be performed each week:

1. Each week, check the “AMB Find Episode – Anticoag Antiplatelet GI Risk – Clinician Notification Only” workbench report (report ID: 12465957) to identify patients who may benefit from intervention. This list will be populated at the beginning of each week with new patients who require intervention during the coming week. It will also show patients from previous weeks who have not yet made a medication change and remain at high risk for upper GI bleeding.
2. Identify the “target clinician” who should receive the notification message. If the patient has a cardiologist listed in the “last appt provider” column of the report, this indicates that the patient has seen the named cardiologist in the last 12 months, and this will be the target clinician. If the “last appt provider” column is blank, the responsible provider on record (indicated in the “responsible provider(s)” column) will be the target clinician.
3. Open an anticoag encounter for the patient using the “anticoag encounter” button at the top of the workbench report and select “letter from anticoagulation” as the reason for the visit.
4. Create a new letter using the blank letter template and insert the smarttext “AMB ANTICOAG GI RISK” (ID: 2100720185) into the letter.
   1. Fill in any wildcards in the letter template, mark the letter as high priority, and route the letter to the clinician identified as the target clinician in step 2.
5. Going back into the workbench report, look at the column “send patient activation guide?”.
   1. If there is a YES in this column, go back into the anticoag encounter and draft a second letter to the patient using the smarttext “AMB ANTICOAG GI PT ACTIVATION BROCHURE” (2100720187)
      1. In the smarttext letter template, fill in the name of the target clinician that you routed the anticoag encounter letter to in step 4 above along with the phone number for the clinic that the patient sees this clinician at.
         - You can find the clinic phone number by navigating to the patient’s last visit with this clinician and copying the phone number for the clinic. If you are unable to locate the clinic phone number for the target provider, enter the main hospital line in this wildcard field (734-936-4000).
   2. If there is a NO in this column, continue to step 6.
6. The clinician notification letter requests that the clinician respond with their plan of care for the patient by routing the letter back to the nurse.
7. Upon receiving a response from the clinician documenting their plan of care, the nurse can sign and close the encounter.
8. **CONTINGENCY PLANS FOR UNEXPECTED COMMUNICATIONS FROM PATIENTS OR CLINICIANS**

- If the patient contacts the anticoagulation clinic with questions related to the upper GI bleeding risk reduction project or their GI bleeding risk, direct the patient to speak with the clinician who received the clinician notification message.
- If the clinician responds to the message stating that they have not seen the patient recently or that they do not believe they are the best clinician to decide regarding medication optimization for the patient, redirect the message to the responsible provider on record for managing the patient’s warfarin therapy.
- If the patient has concerns or questions about proton pump inhibitors (PPIs), the smartphrase “.startppi” has language you can use to answer common questions patients may have.
- If clinicians are unsure about a medication change or have questions about whether either strategy proposed in the clinician notification is appropriate, remind the clinician that the most appropriate plan of care is different for each patient and ask them to use their best clinical judgment in deciding to lower the patient’s risk of bleeding.

1. **REFERENCES:**

Bhatt DL, Scheiman J, Abraham NS, et al. ACCF/ACG/AHA 2008 expert consensus document on reducing the gastrointestinal risks of antiplatelet therapy and NSAID use: a report of the American College of Cardiology Foundation Task Force on Clinical Expert Consensus Documents. *J Am Coll Cardiol*. 2008;52(18):1502-1517. doi:10.1016/j.jacc.2008.08.002

1. **AUTHOR(S):**

Jacob E. Kurlander, MD, MS

Geoffrey D. Barnes, MD, MSc

Danielle Helminski, MPH

Linda K. Perry, BSN, RN

Jackie Parsons, BSN, RN

1. **PROCESS FLOWCHART:**
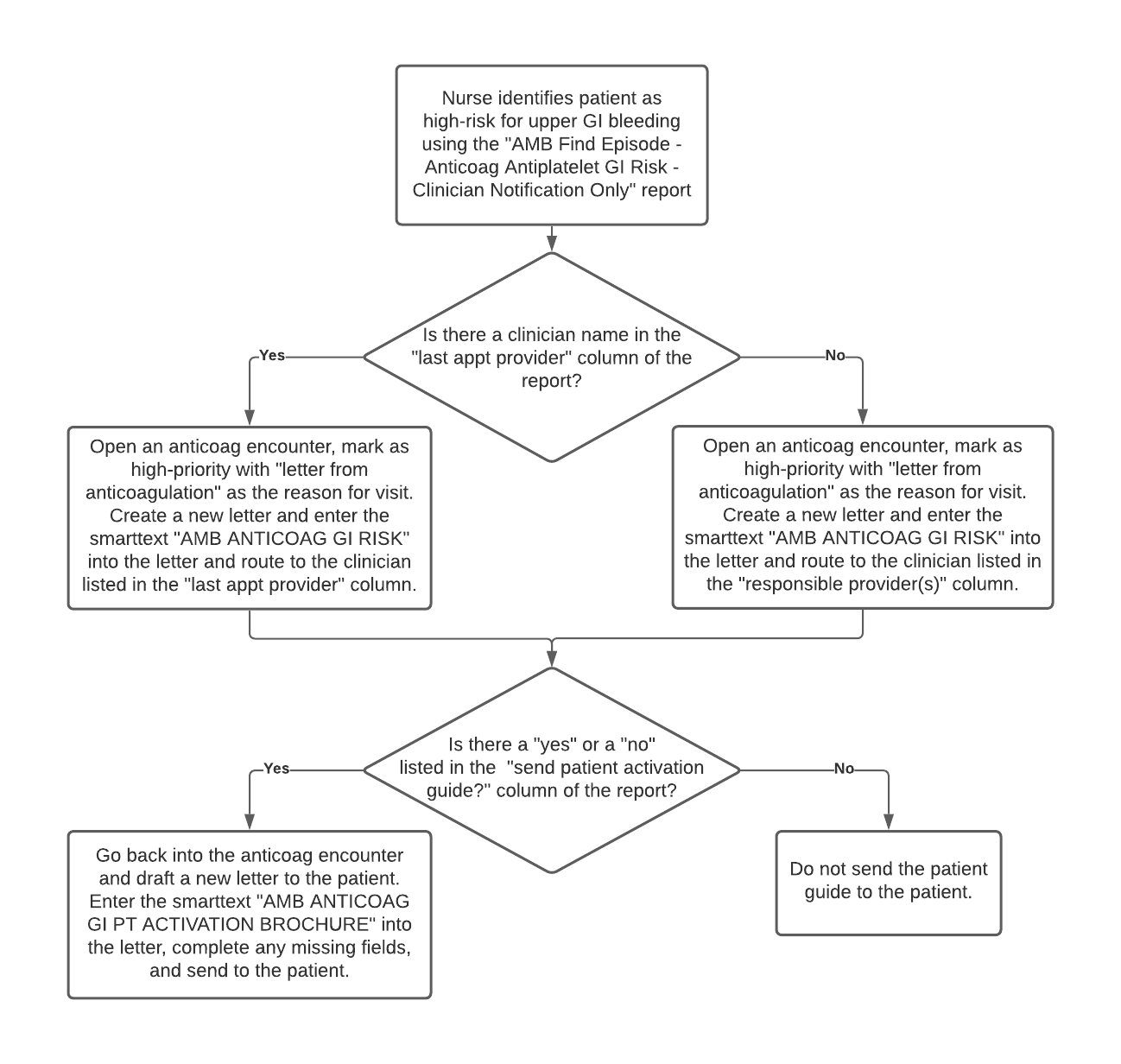


## Clinician Notification + Nurse Facilitation Clinic Staff Protocol for RNs

**Anticoagulation Service**

**Protocol for Upper GI Bleeding Reduction Project**

**Clinician Notification + Nurse Facilitation Intervention**

1. **DEFINITION:**

Patients are designated as high risk for upper gastrointestinal (GI) bleeding if they use warfarin along with aspirin (either 325mg or 81mg) and/or a thienopyridine (clopidogrel, prasugrel, ticagrelor) without a proton pump inhibitor (PPI).

1. **PURPOSE:**

This is a quality improvement initiative to help clinicians take appropriate steps to reduce upper GI bleeding risk for patients on warfarin and an antiplatelet drug. As part of this project, a new patient education guide is being trialed. This protocol defines the role of the anticoagulation clinic nurse in this project.

1. **PATIENT ELIGIBILITY:**

All Michigan Medicine Anticoagulation Service patients who are high risk for upper GI bleeding (as defined above) and who are anticipated to use warfarin for at least 90 days. Patients with an LVAD, heart transplant, or allergy/intolerance to PPI will be excluded.

1. **PROCEDURE:**

The following steps should be performed each week:

1. Each week, check the “AMB Find Episode – Anticoag Antiplatelet GI Risk – Clinician Notification and Nurse facilitation” workbench report (report ID: 12465958) to identify patients who may benefit from intervention. This list will be populated at the beginning of each week with new patients who require intervention during the coming week. It will also show patients from previous weeks who have not yet made a medication change and remain at high risk for upper GI bleeding.
2. Identify the “target clinician” who should receive the notification message. If the patient has a cardiologist listed in the “last appt provider” column of the report, this indicates that the patient has seen the named cardiologist in the last 12 months, and this will be the target clinician. If the “last appt provider” column is blank, the responsible provider on record (indicated in the “responsible provider(s)” column) will be the target clinician.
3. Open an anticoag encounter for the patient using the “anticoag encounter” button at the top of the workbench report and select “letter from anticoagulation” as the reason for the visit.
4. Create a new letter by selecting “create new communication” then selecting “other” and searching for the smarttext “AMB ANTICOAG GI RISK CNNF RN” (ID: 2100720186) then entering it into the letter.
   1. Complete chart review for the patient and enter the indication for antiplatelet therapy into the report. If there is more than one indication for antiplatelet therapy, indicate all of them in the dropdown. Fill in any additional wildcards in the letter template, mark the letter as high priority, and route the letter to the clinician identified as the target clinician in step 2.
5. Going back into the workbench report, look at the column “send patient activation guide?”.
   1. If there is a YES in this column, go back into the anticoag encounter and draft a new letter to the patient using the smarttext “AMB ANTICOAG GI PT ACTIVATION BROCHURE” (2100720187)
      1. In the smarttext letter template, fill in the name of the target clinician that you routed the anticoag encounter letter to in step 4 above along with the phone number for the clinic that the patient sees this clinician at.
         1. You can find the clinic phone number by navigating to the patient’s last visit with this clinician and copying the phone number for the clinic. If you are unable to locate the clinic phone number for the target provider, enter the main hospital line in this wildcard field (734-936-4000).
   2. If there is a NO in this column, continue to step 6.
6. The clinician notification letter requests that the clinician respond with their plan of care for the patient by routing the letter back to the nurse.
7. Upon receiving a response from the clinician documenting their plan of care, the RN should facilitate the medication change and patient education recommended by the clinician in their response.
   1. If the clinician recommends initiation of a PPI for gastroprotection, pend the order for the PPI in MiChart for the clinician to sign off on.
   2. If the clinician recommends discontinuation of antiplatelet therapy, remove the patient’s antiplatelet drug from their current medication list in MiChart.
8. Contact the patient by phone to communicate the clinician’s plan of action and provide education about the medication change to the patient and answer any questions that the patient may have (the smarttext “.startppi” has common patient questions and answers available to help guide this discussion).
   1. Document that the call was completed, and the medication change was communicated to the patient by sending a letter to the patient after the call has been completed using the appropriate smarttext for the medication change recommended:
      1. “AMB ANTICOAG CNNF START PPI” (ID: 775446) – to be used when clinician recommends patient start a PPI
      2. “AMB ANTICOAG CNNF STOP APT” (ID: 775456) – to be used when clinician recommends patient stop their antiplatelet drug
9. Once any recommended medication change has been communicated to the patient and patient education has been provided and documented in MiChart, the nurse can sign and close the encounter.
10. **CONTINGENCY PLANS FOR UNEXPECTED COMMUNICATIONS FROM PATIENTS OR CLINICIANS**

- If the patient contacts the anticoagulation clinic with questions related to the upper GI bleeding risk reduction project or their GI bleeding risk, direct the patient to speak with the clinician who received the clinician notification message.
- If the clinician responds to the message stating that they have not seen the patient recently or that they do not believe they are the best clinician to decide regarding medication optimization for the patient, redirect the message to the responsible provider on record for managing the patient’s warfarin therapy.
- If the patient has concerns or questions about proton pump inhibitors (PPIs), the smartphrase “.startppi” has language you can use to answer common questions patients may have.
- If clinicians are unsure about a medication change or have questions about whether either strategy proposed in the clinician notification is appropriate, remind the clinician that the most appropriate plan of care is different for each patient and ask them to use their best clinical judgment in deciding to lower the patient’s risk of bleeding.

1. **REFERENCES:**

Bhatt DL, Scheiman J, Abraham NS, et al. ACCF/ACG/AHA 2008 expert consensus document on reducing the gastrointestinal risks of antiplatelet therapy and NSAID use: a report of the American College of Cardiology Foundation Task Force on Clinical Expert Consensus Documents. *J Am Coll Cardiol*. 2008;52(18):1502-1517. doi:10.1016/j.jacc.2008.08.002

1. **AUTHOR(S):**

Jacob E. Kurlander, MD, MS

Geoffrey D. Barnes, MD, MSc

Danielle Helminski, MPH

Linda K. Perry, BSN, RN

Jackie Parsons, BSN, RN

1. **PROCESS FLOWCHART:**


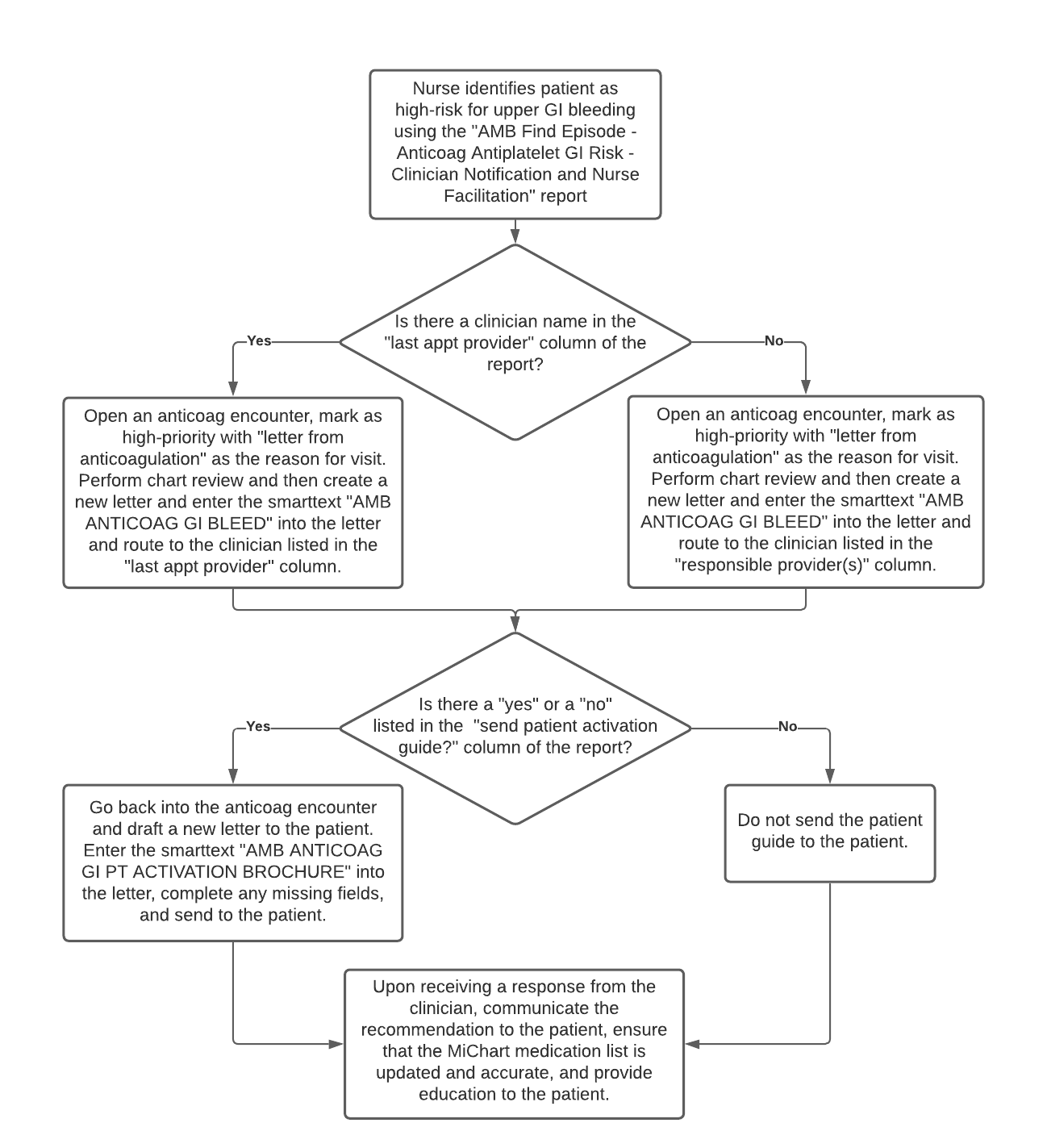

Supplement: Supplementary file 10 — Additional file 10: Supplement 10. Anticoagulation Nurse ProtocolsR0.docx [file 43058_2022_256_MOESM10_ESM.docx]
