# Supplementary material for: Development of a multicomponent implementation strategy to reduce upper gastrointestinal bleeding risk in patients using warfarin and antiplatelet therapy, and protocol for a pragmatic multilevel randomized factorial pilot implementation trial
Source: Implement Sci Commun. 2022 Jan 28;3:8. doi: 10.1186/s43058-022-00256-8 (PMC8796614; doi:10.1186/s43058-022-00256-8)
Supplement: Supplementary file 11 — Additional file 11: Supplement 11. Exploratory OutcomesR0.docx [file 43058_2022_256_MOESM11_ESM.docx]

# **Supplement 11.** Exploratory Outcomes

## **Table 1.** Exploratory Objectives and Endpoints

| **OBJECTIVES** | **ENDPOINTS** |
| --- | --- |
| To explore additional aspects of the feasibility of recruitment. | Proportion of randomized patients able to be reached by phone after three attempts for patient assessment #1 at week 5.    Average number of phone call attempts to reach each patient for patient assessment #1 at week 5.    The number of anticoagulation clinic patients who meet eligibility criteria for the AEGIS trial on the first day of the pilot study according to the EHR workbench report. |
| To explore changes in the proportion of eligible patients during the study duration. | The proportion of all patients in the anticoagulation clinic who meet eligibility criteria for the study, regardless of whether they are included, who discontinued warfarin or were otherwise closed to the anticoagulation service during the study period.  The proportion of patients who were randomized who discontinued warfarin or were closed to the anticoagulation clinic service during the participants’ study duration. |
| To explore the accuracy of the electronic health record’s medication list for ascertaining inclusion criteria | The proportion of randomized patients who, retrospectively, report that they had been using antiplatelet therapy at baseline during patient assessment #1 at week 5.    The proportion of randomized patients who, retrospectively, report that they had been using PPI at baseline during patient assessment #1 at week 5.  The proportion of randomized patients who met inclusion criteria based on retrospective medication use in patient assessment #1 at week 5. |
| To explore the accuracy of the electronic health record’s medication list for ascertaining medication changes | The accuracy, positive predictive value, and negative predictive value of the electronic health record for use of antiplatelet therapy, and for use of PPIs at the time of assessment #3.  The accuracy, positive predictive value, and negative predictive value of the electronic health record for “medication optimization” at the time of assessment #3. A patient will be considered to have had medication optimization if they are either no longer using combination antithrombotic therapy or are using a PPI. |
| To explore the feasibility of complete collection of study data | The proportion of patients with complete data entry in RedCAP database at study completion  Average duration of interviews on medication use/adherence during patient assessment #3 at week 9-12. |
| To explore the feasibility of delivering the clinician-level interventions | Proportion of patients whose clinician was sent the clinician-level intervention by the anticoagulation clinic staff in the prescribed time period per the clinic protocol. We will also separately calculate this endpoint for the two levels of the clinician intervention. |
| To explore the feasibility of delivering the patient activation tool | The proportion of patients randomized to receive the activation guide who were sent the guide in the prescribed period of time, as determined by chart review.    The proportion of patients randomized to receive the guide who recalled receiving the guide during patient assessment #1 at week 5.  The proportion of patients randomized to receive the guide who recalled reviewing the guide in patient assessment #1 at week 5. |
| To explore the feasibility of prompting patient-clinician communication about medication optimization | The number of days after delivery of the provider intervention at which the patient and the target clinician (or their healthcare team) had communication (either by phone, in person visit, or portal message) about medication optimization, based on chart review. This will be calculated overall and separately for each intervention arm.  The proportion of patients who had a communication about medication optimization with their clinicians based on patient recall at week 5 (patient assessment #1) and at week 9-12 (patient assessment #3). This will be calculated overall and separately for each intervention arm.  For patients who had a communication with their clinician about medication optimization, the proportion in whom initial contact was made by the clinician vs. by the patient, according to chart review. This will be calculated overall and separately for each of the intervention arms. This will be assessed based on week 5 call.  For patients who had a communication with their clinician about medication optimization, the proportion in whom initial contact was made by the clinician vs. by the patient, according to patient recall. This will be calculated overall and separately for each intervention arm.  For patients who contacted a clinician about medication optimization, the specialty of the clinician and whether the clinician who was sent the clinician intervention was the target clinician. This will be determined separately based on chart review and patient recall.  For patients who had communication with their clinician documented in the electronic health record, whether this occurred by phone, portal, or in-person/telehealth visit based on chart review. |
| To explore process measures associated with clinician notification | **For clinicians randomized to clinician notification:**    Number of days taken for clinicians to send a response message to anticoagulation staff after first receiving the clinician intervention.  Proportion of clinicians who documented their plan-of-care for the patient in the response message to the anticoagulation staff.  The number of clinicians who appropriately documented changes in antiplatelet drugs or PPI in the EHR medication list. |
| To explore process outcomes associated with the clinician notification + nurse facilitation implementation strategy | **For clinicians and patients randomized to clinician notification + Nurse Facilitation:**  Number of days taken for clinicians to send a response message to anticoagulation staff after first receiving the clinician reminder.  Proportion of clinicians who documented their plan-of-care for the patient in the response message to the anticoagulation staff.  Proportion of patients randomized to clinician notification + nurse facilitation for whom the anticoagulation nurse provided patient education.  Proportion of patients randomized to clinician notification + nurse facilitation for whom the anticoagulation nurse pended the PPI order. |
| To explore aspects of effectiveness of the implementation strategies. | For anticoagulation clinic patients who meet inclusion criteria but are not randomized to an intervention arm in the pilot trial, whether or not they initiate PPI or discontinue antiplatelet therapy over the study period, as determined using electronic health record data, which will provide data on use of the evidence-based practices in usual care.    For randomized patients, initiation of either a PPI or discontinuation of all antiplatelet therapy at week 5 (patient assessment #1), and at week 9-12 (patient assessment #3), as determined by patient interview.    For randomized patients, the level of adherence to the recommended medication change (either PPI initiation or antiplatelet discontinuation) at week 9-12 measured using the Wilson questionnaire, as determined by patient assessment #3.    Documentation of a recommendation by one of the patient’s Michigan Medicine clinicians to discontinue antiplatelet therapy or initiate a PPI as indicated by a clinical documentation or by a change in the EHR medication list, ascertained by EHR review. |
| To explore the appropriateness of antiplatelet therapy used by patients at the time of study randomization. | The clinical appropriateness of antiplatelet therapy at the time of study initiation, classified as either (probably guideline concordant, probably not guideline concordant, or uncertain), based on detailed chart review and in reference to practice recommendations (Supplement 6). |
| To explore the appropriateness of medication changes in antiplatelet therapy during the trial. | For patients who either stopped antiplatelet therapy or initiated a PPI during the trial, to explore the appropriateness of antiplatelet management according to patient-reported use in patient assessment #3 at week 9-12. The clinical appropriateness of antiplatelet management will be determined according to pre-specified criteria (probably guideline concordant, probably not concordant, uncertain). See supplement 6 for criteria. |
